# Wolf in sheep's clothing: Model misspecification undermines tests of the neutral theory for life histories

**DOI:** 10.1002/ece3.2874

**Published:** 2017-04-04

**Authors:** Matthieu Authier, Lise M. Aubry, Emmanuelle Cam

**Affiliations:** ^1^ Observatoire PELAGISUMS‐CNRS 3462Université de la RochelleLa RochelleFrance; ^2^ Wildland Resources Department & the Ecology CenterUtah State UniversityLoganUTUSA; ^3^ Laboratoire Évolution & Diversité BiologiqueUMR 5174Université Toulouse IIICNRSENSFEAIRD, Toulouse Cedex 9France

**Keywords:** evolutionary ecology, heterogeneity, life history, misspecification, neutral model, null model, state dependence

## Abstract

Understanding the processes behind change in reproductive state along life‐history trajectories is a salient research program in evolutionary ecology. Two processes, state dependence and heterogeneity, can drive the dynamics of change among states. Both processes can operate simultaneously, begging the difficult question of how to tease them apart in practice. The Neutral Theory for Life Histories (NTLH) holds that the bulk of variations in life‐history trajectories is due to state dependence and is hence neutral: Once previous (breeding) state is taken into account, variations are mostly random. Lifetime reproductive success (LRS), the number of descendants produced over an individual's reproductive life span, has been used to infer support for NTLH in natura. Support stemmed from accurate prediction of the population‐level distribution of LRS with parameters estimated from a state dependence model. We show with Monte Carlo simulations that the current reliance of NTLH on LRS prediction in a null hypothesis framework easily leads to selecting a misspecified model, biased estimates and flawed inferences. Support for the NTLH can be spurious because of a systematic positive bias in estimated state dependence when heterogeneity is present in the data but ignored in the analysis. This bias can lead to spurious positive covariance between fitness components when there is in fact an underlying trade‐off. Furthermore, neutrality implied by NTLH needs a clarification because of a probable disjunction between its common understanding by evolutionary ecologists and its translation into statistical models of life‐history trajectories. Irrespective of what neutrality entails, testing hypotheses about the dynamics of change among states in life histories requires a multimodel framework because state dependence and heterogeneity can easily be mistaken for each other.

## 1 Introduction

An observed life history is the integrative result of an individual's ability to grow, survive, and reproduce (Reznick, Nunney, & Tessier, 2000). Standing at the crossroads of demography and evolutionary ecology, life‐history studies focus on how individuals of a given generation manage to spread their genes into the next (Metcalf & Parvard, 2007). A salient line of inquiry seeks to explain the interindividual variability in life histories of iteroparous organisms in the wild (Cam, Aubry, & Authier, 2016). This topic has sustained a steady number of new publications (≈20 every year since 2010) and a large number of citations (*>*300 per year) in the ecological research community over the past 5 years (Appendix S1). An important question is to what extent, if any, are variations in life histories heritable. Individual heterogeneity is the oft‐used term to explain variation in life‐history traits, variations which can fuel adaptive phenotypic evolution (Wilson & Nussey, 2010). By contrast, individual stochasticity refers to variations that are irrelevant to natural selection: “[t]he movement of an individual through its life cycle is a random process, and although [death] is certain, the pathways taken to that destination are stochastic and will differ even between identical individuals…” (Caswell, 2009). Individual stochasticity sensu Caswell (2009) manifests itself in the diversity of life‐history trajectories: Would the same individual be able to live its life a second time; the trajectory would be different simply because of sampling variation. What causes these different trajectories is at the core of recent studies debating the relative importance of within‐ and between‐individual variance in life histories, and in particular, whether observed variations are selectively neutral or not (Bonnet & Postma, 2016; Cam et al., 2016; 2013; Jenouvrier, Péron, & Weimerskirch, 2015; Plard, Bonenfant, Delorme, & Gaillard, 2012; Steiner & Tuljapurkar, 2012).

Two mechanisms can explain how variations in individual trajectories may arise (see Cam et al., 2016 for a review): (1) state dependence and (2) heterogeneity. True state dependence sensu Heckman (1981) is the process whereby “past experience has a genuine behavioral effect in the sense that an otherwise identical individual who did not experience the event would behave differently in the future than an individual who experienced the event.” Although originally framed in the context of human behavior, this definition is not restrictive but could include other processes (e.g., physiology). An event means the realization of a random variable such as successful breeding. State dependence describes a Markovian process in which experiencing an event affects an individual and changes its propensity to re‐experience the event. State dependence can generate variation (also known as “dynamic heterogeneity”) in a population of identical individuals, simply because of sampling variance in the realization of stochastic processes such as survival or reproduction (Caswell, 2009; Orzack, Steiner, Tuljapurkar, & Thompson, 2011; Steiner & Tuljapurkar, 2012; Steiner, Tuljapurkar, & Orzack, 2010; Tuljapurkar, Steiner, & Orzack, 2009). This sampling variance is a within‐individual variance or “individual stochasticity” sensu Caswell (2009).

In contrast, the heterogeneity hypothesis starts from the concern that all relevant variables that can affect an individual's fate may not be available to the investigator, either because they are unknown, difficult to measure or not directly observable (Mood, 2010; Wienke, 2010). Assuming this heterogeneity is fixed (time‐invariant), it is hidden to investigators but may account for the correlation between states in the life‐history trajectory of a given individual. Cam et al. (2016) speak of hidden permanent demographic heterogeneity (HPDH). HPDH statistically translates into a between‐individual variance due to unobserved differences at the individual level, upon which natural selection may act if individual variation is heritable (Chambert, Rotella, & Garrott, 2014; Wilson & Nussey, 2010). HPDH is commonly estimated with generalized linear mixed models (Bolker et al., 2009) or mixture models (Fay, Barbraud, Delord, & Weimerskirch, 2016): It corresponds to “individual quality” (Bergeron, Baeta, Pelletier, Réale, & Garant, 2011; Cam et al., 2016; Wilson & Nussey, 2010). HPDH does not exclude random variation, but relies on statistical models to partition the variance in individual trajectories into between‐individual and within‐individual components (Van de Pol & Wright, 2009). Such variance‐partitioning models have been heavily used, in part because they address pseudo‐replication when measurements from the same individual are still correlated after accounting for observed covariates. However, recent studies drew attention to the theoretical implications of taking HPDH for granted in the analysis of life‐history evolution (Orzack et al., 2011; Steiner & Tuljapurkar, 2012; Steiner et al., 2010; Tuljapurkar et al., 2009).

Both state dependence and HPDH are concerned with accounting for changes in states along an individual's life‐history trajectory. With HPDH, any change in state is short lived and an individual quickly returns to a trajectory reflecting its latent “quality”. This results in repeatability in one state (success or failure) with short‐lived visits to the other state. With state dependence, change can be more sustained in the case of positive state dependence, or short lived in the case of negative state dependence (trade‐off). In other words, positive state dependence leads to some degree of persistence in state, where past experience of failure (for example) increases the probability of experiencing failure again. This can be illustrated by the “spiral of failure” phenomenon in behavioral ecology, whereby breeding failure is associated with increased probabilities of dispersing and divorcing, both being in turn associated with increased probability of unsuccessful reproduction in the following year (Naves, Monnat, & Cam, 2006). The current controversy thus revolves around the evolutionary significance of life‐history variations or what drives intra‐ and interindividual changes in life‐history outcomes: Is it mostly due to state dependence, HPDH, or a combination of both? In other words, what are the relative fractions of neutral and potentially non‐neutral variations in life histories?

This question motivated the development of a Neutral Theory for Life Histories (hereafter NTLH, e.g*.,* Orzack et al., 2011; Steiner & Tuljapurkar, 2012; Steiner et al., 2010; Tuljapurkar et al., 2009). NTLH studies concluded that the bulk of variations in life histories observed in natura across a wide panel of species was selectively neutral, a result that took empiricists by surprise and called for renewed vigilance against adaptationism (Gould & Lewontin, 1979; Pigliucci & Kaplan, 2000). NTLH investigations take a Markovian model with state dependence as an appropriate null model. This null model is deemed neutral because it does not include HPDH: All individuals are assumed to have the same phenotype (Steiner & Tuljapurkar, 2012). Parameters of this null model are estimated from data and subsequently used to predict the distribution of lifetime reproductive success (LRS). Lifetime reproductive success is an individual‐level metric: It is the number of descendants an individual produces over its reproductive life span, conditional on the individual having recruited into the breeding population. As a measure of individual fitness, the shortcomings of LRS are well known (Metcalf & Parvard, 2007), although this does not prevent its use in practice (e.g. Mourocq et al., 2016). Lifetime reproductive success, which was used extensively in testing for the presence of HPDH in NTLH studies (Bonnet & Postma, 2016), is scrutinized at the population level (Tuljapurkar et al., 2009). If the predicted (population‐level) distribution of LRS matches the observed one, support for the NTLH is inferred. Quoting Tuljapurkar et al. (2009): “[State dependence] can provide a ‘neutral’ standard by which one can assess whether the observed distribution of fitness components, such as the LRS or average annual reproduction, are influenced by certain kinds of [HPDH]. In particular, a lack of fit between an observed distribution of, say, the LRS and a distribution generated solely by dynamic heterogeneity [i.e., state dependence] suggests that the observed distribution is influenced by fixed differences among individuals.” This statement is normative about the ability and usefulness of LRS to infer HPDH in life histories: If a good fit is obtained between the observed distribution of LRS and the one predicted from parameter estimates following model fitting, then this model is likely to provide a good approximation to the true data‐generating mechanism.

This statement has, however, not been empirically evaluated. In other words, can a data‐generating mechanism that involves only HPDH predict a population‐level LRS distribution that is identical to one expected from a data‐generating mechanism that involves only state dependence? Can the current NTLH methodology lead to model misspecification? Model misspecification happens when data are analyzed with, and inferences drawn from a model that is very different from the true data‐generating mechanism (Burnham & Anderson, 2002:158). Previous tests of NTLH (e.g*.,* Orzack et al., 2011; Steiner & Tuljapurkar, 2012; Steiner et al., 2010; Tuljapurkar et al., 2009) implicitly assumed that model misspecification has no impact on parameter estimation.

We empirically explore this premise with Monte Carlo simulations, using LRS and entropy as in the standard NTLH framework (Tuljapurkar et al., 2009). We compare models including no HPDH and no state dependence, state dependence only, HPDH only, and both state dependence and HPDH. Our focus is on accurate estimation of parameters in statistical models of life histories: This study thus complements the power analysis of Bonnet and Postma (2016). Simulations generate data according to a known process, hereafter referred to as the true data‐generating mechanism. Knowledge of the true value of parameters enabled to assess bias.

## 2 Assumptions

For data simulation, we made the following assumptions. Individuals have recruited into the breeding population and survived to a second breeding occasion: The shortest breeding trajectory includes two occasions. Once recruited, no individual skips breeding, but there is (Bernoulli) variability in breeding success. HPDH can be described by a bivariate normal distribution with possible correlation between individual survival and breeding success propensities.

We simulated both survival and breeding trajectories to mimic data commonly used to investigate HPDH (e.g., Cam, Link, Cooch, Monnat, & Danchin, 2002; Cam et al., 2013). Only simulated data on reproductive life histories were subsequently analyzed, but survival allowed us to take into account that reproductive life span is a random variable. We chose a mean survival (conditional on having recruited in the breeding segment) of ϕ = 0.75 and simulated 1*,*000 individual life histories of maximum length 42. These values were chosen to reflect the biology of long‐lived iteroparous organisms, such as black‐legged kittiwakes (*Rissa tridactyla*) about which conflicting results on NTLH have been published (Cam et al., 2013; Steiner et al., 2010). Across simulation scenarios, the expected sample size is 1,000 × $\left(1+\sum_{k=0}^{40}\phi^{k}\right)$ ≈ 5,000. This is a large sample size, both with respect to the number of individual trajectories, and their length: Asymptotic justifications of statistical tests should hold.

## 3 Notations

Greek letters denote the true value of a parameter, which is unknown in analyses of empirical data. Greek letters with a hat denote estimated parameters from a model and data. Let ${\text{survival}}_{it}$ denotes the survival of individual *i* in year *t*:

(1) $${\text{survival}}_{it}∼\text{Bernoulli}\left({\text{logit}}^{-1}\left(\mu_{\phi }+\alpha_{i,1}\right)\right)$$

where $\mu_{\phi }=\text{logit}\left(\phi \right)=\text{log}\left(\phi /1-\phi \right)$, and ϕ is the average survival probability. Conditional on ${\text{survival}}_{it}=1$, individual *i* breeds in year *t* with success probability $p_{it}$:

(2) $${\text{success}}_{it}|{\text{survival}}_{it}∼\text{Bernoulli}\left(p_{it}={\text{logit}}^{-1}\left(\mu +\alpha_{i,2}+\gamma \times {\text{success}}_{i(t-1)}\right)\right)$$

where μ is an intercept, γ is the parameter quantifying state dependence, and $\left(\alpha_{i,1},\;\alpha_{i,2}\right)$ are individual random effects:

(3) $$\left(\begin{array}{c} \alpha_{i,1}\\ \alpha_{i,2} \end{array}\right)∼\;N_{2}\left(\;\begin{array}{c} 0\\ 0 \end{array},\;\left[\begin{array}{cc} \sigma_{\phi }^{2} & \mathrm{cor}\times \sigma_{\phi }\times \sigma_{\mathrm{repro}}\\ \mathrm{cor}\times \sigma_{\phi }\times \sigma_{\mathrm{repro}} & \sigma_{\mathrm{repro}}^{2} \end{array}\right]\right)$$

$N_{2}$ denotes a bivariate normal distribution. The parameters $\sigma_{\phi }^{2}$ and $\sigma_{\mathrm{repro}}^{2}$ are variance parameters, and cor is a correlation parameter bounded between −1 and 1. The multivariate normal distribution was used for convenience as often in empirical investigations (e.g., Authier, Cam, & Guinet, 2011; Browne, McCleery, Sheldon, & Pettifor, 2007; Cam et al., 2013).

### 3.1 Between‐individual Variations

Equation 3 reflects the idea of a heterogeneous population: Individuals have different phenotypes and can be ranked along a continuous gradient of propensity to survive and breed with success. The parameters $\sigma_{\phi }$ (also known as frailty; Wienke, 2010; Wintrebert, Zwinderman, Cam, Pradel, & van Houwelingen, 2005) and $\sigma_{\mathrm{repro}}$ quantify HPDH (at the population‐level) in the survival and breeding processes, respectively. They reflect unmeasured between‐individual differences while cor is the correlation between individual survival and breeding success propensities. A negative correlation corresponds to an individual‐level trade‐off between survival and breeding success, while a positive correlation corresponds to the reverse situation of “individual quality” whereby successful breeders survive best (Cam et al., 2002).

### 3.2 State dependence

γ is the parameter quantifying state dependence, that is the succession of 0s (failures) and 1s (successes) in the breeding trajectory. A causal interpretation of γ follows from Heckman, 1981's counterfactual definition: “an otherwise identical individual who did not experience the event would behave differently in the future than an individual who experienced the event.” If an individual *i* successfully bred at time *t *−* * 1, the probability of breeding with success again at time *t* is:

(4) $$\log\left(\frac{p_{it}}{1-p_{it}}\right)=\mu +\alpha_{i,2}+\gamma$$

The counterfactual probability $p_{it}^{*}$ corresponds to what would be the probability of breeding successfully at time *t* had individual *i* failed at time *t* − 1:

(5) $$\log\left(\frac{p_{it}^{*}}{1-p_{it}^{*}}\right)=\mu +\alpha_{i,2}$$

Subtracting Equation 5 from Equation 4 yields a general definition of γ:

(6) $$\gamma =\log\left(\frac{p_{it}}{1-p_{it}}\right)-\log\left(\frac{p_{it}^{*}}{1-p_{it}^{*}}\right)=\log\left(\frac{\frac{p_{it}}{1-p_{it}}}{\frac{p_{it}^{*}}{1-p_{it}^{*}}}\right)=\log\left(\frac{p_{it}\left(1-p_{it}^{*}\right)}{p_{it}^{*}\left(1-p_{it}\right)}\right)$$

γ is the log odds ratio (*OR*) of how much having experienced the event (${\text{success}}_{i(t-1)}=1$) affects the probability of experiencing it again relative to not having experienced it (${\text{success}}_{i(t-1)}=0$). For example, an odds ratio of 2 means that a successful breeder is twice more likely to breed successfully again compared to a failed one. Likewise, an odds ratio of ½ means that a successful breeder is half as likely to breed successfully again compared to a failed one. An odds ratio less than 1 (γ<0) would be evidence of a cost of reproduction, and a trade‐off between current and future reproduction. The interpretation of γ as an odds ratio is convenient for simulating realistic amounts of state dependence. γ is assumed the same for all individuals.

### 3.3 Entropy

The transition matrix $\Psi_{i}$ describes how an individual *i* that survived from *t*−1 to *t* can change states:

(7) $$\Psi_{i}=\left[\begin{array}{cc} \psi_{i}^{11} & \psi_{i}^{12}\\ \psi_{i}^{21} & \psi_{i}^{22} \end{array}\right]=\left[\begin{array}{cc} 1-p_{it}^{*} & p_{it}^{*}\\ 1-p_{it} & p_{it} \end{array}\right]$$

where $\psi_{i}^{11}$ is the probability of a failed breeder to fail again, $\psi_{i}^{12}$ is the probability of a failed breeder to become successful, $\psi_{i}^{21}$ is the probability of a successful breeder to fail its next breeding attempt, and $\psi_{i}^{22}$ is the probability of a successful breeder to breed successfully again.

The average entropy, which measures randomness in transitions between states, of the transition matrix $\psi_{i}$ is:

(8) $$H_{i}=-\pi_{1}\times \left(\psi_{i}^{11}\text{log}\psi_{i}^{11}+\psi_{i}^{12}\text{log}\psi_{i}^{12}\right)-\pi_{2}\times \left(\psi_{i}^{21}\text{log}\psi_{i}^{21}+\psi_{i}^{22}\text{log}\psi_{i}^{22}\right)$$

where $\pi^{\prime }=\left(\pi_{1},\;\pi_{2}\right)$ are the stationary proportions of failures and successes along trajectories (Tuljapurkar et al., 2009). Equation 8 can be written as:

(9) $$\begin{array}{cc} H_{i}= & -\frac{\psi_{i}^{21}}{\psi_{i}^{21}+\psi_{i}^{12}}\times \left(\psi_{i}^{11}\text{log}\psi_{i}^{11}+\psi_{i}^{12}\log\psi_{i}^{12}\right)\\ & -\frac{\psi_{i}^{12}}{\psi_{i}^{21}+\psi_{i}^{12}}\times \left(\psi_{i}^{21}\text{log}\psi_{i}^{21}+\psi_{i}^{22}\text{log}\psi_{i}^{22}\right) \end{array}$$

that is,

(10) $$\begin{array}{cc} H_{i}= & -\frac{1-p_{it}}{1-p_{it}+p_{it}^{*}}\times \left(\left(1-p_{it}^{*}\right)\text{log}\left(1-p_{it}^{*}\right)+p_{it}^{*}\text{log}p_{it}^{*}\right)\\ & -\frac{p_{it}^{*}}{1-p_{it}+p_{it}^{*}}\times \left(\left(1-p_{it}\right)\text{log}\left(1-p_{it}\right)+p_{it}\text{log}p_{it}\right) \end{array}$$

### 3.4 Within‐individual Variations

Individual stochasticity sensu Caswell (2009) is a sampling variance, or within‐individual variance in states, here successful versus failed breeding attempt: “[t]he variance in the [states] is the result of luck, not heterogeneity.” For a Bernoulli trial with success probability π, the sampling variance in observed outcomes is given by the formula π×(1−π). For an average individual *i (α*_*i*,2_ = 0 in Equation 2), the within‐individual variance in breeding success ($\sigma_{\mathrm{within}}^{2}$) depends on the previous state if true state dependence is operating:

(11) $$\sigma_{\mathrm{within}}^{2}=\left\{\begin{array}{c} p_{it}\times \left(1-p_{it}\right)=\frac{1}{2+e^{\mu +\gamma }+\;e^{-(\mu +\gamma )}}{\text{if success}}_{i(t-1)}=1;\\ P_{it}^{*}\times \left(1-p_{it}^{*}\right)=\frac{1}{2+e^{\mu }+\;e^{-\mu }}{\text{if success}}_{i(t-1)}=0. \end{array}\right.$$

This within‐individual variance is different from that of Steiner et al. (2010), which refers to variations solely generated by the stochastic nature of the transitions in reproductive stages (page 439). The variability studied by Steiner et al. (2010) is defined at the level of a trajectory. In practice, the true trajectory that any given individual follows is only known up to that individual's death, which can be purely accidental (e.g*.,* an unfortunate lightning strike). Because death censors a life‐history trajectory, it is impossible to know whether any two individuals that had the same trajectory until their death would have remained on the same trajectory had they both lived longer. It is pragmatically impossible to know whether two individuals are truly sharing the same trajectory. The within‐individual variance in Equation 11 is defined at the individual level for any time step along a realized trajectory. Because variances are additive, the total variance is the sum of all the steps along that trajectory.

All the above equations (Equations 1−11) involved parameters that are unknown in practice and must be estimated from data. With Monte Carlo simulations, the true values of parameters are known: Model misspecification and its impact on parameter estimates (e.g*.,* bias) can be investigated.

## 4 Monte Carlo tudy

### 4.1 Data simulation

We simulated life histories under several scenarios corresponding to different values for the set of 4 parameters $\left(\gamma ,\;\sigma_{\phi },\;\sigma_{\mathrm{repro}},\;\mathrm{cor}\right)$. We considered data‐generating mechanisms with only HPDH $\left(\gamma =0,\;\sigma_{\phi }\neq 0,\;\sigma_{\mathrm{repro}}\neq 0\right)$, only state dependence $\left(\gamma \neq 0,\;\sigma_{\phi }=0,\;\sigma_{\mathrm{repro}}=0\right)$ *,* and with none or both (Table 1). There were 7 × 4 × 4 × 5 = 560 different combinations of values for $\left(\gamma ,\;\sigma_{\phi },\;\sigma_{\mathrm{repro}},\;\mathrm{cor}\right)$. For each combination, 500 random datasets were simulated and analyzed (Appendix S2: Fig. S1‐S2). In all simulation scenarios, the parameter μ was set to 0, corresponding to an average breeding success probability of 0.5.

**Table 1:** Summary of parameter values used in simulations of life history data

Parameter	State dependence (OR scale)	Heterogeneity		Correlation
	*e* ^*γ*^	*σ*_*ϕ*_	*σ*_repro_	cor
None	1	0.01	0.01	0.0
Small	(3/4, 4/3)	0.33	0.33	
Moderate	(3/5, 5/3)	0.66	0.66	±0.3
Large	(1/2, 2/1)	1.00	1.00	±0.6

The magnitude of true state dependence is given on an odd ratio (OR) scale: Values above 1 (below 1) correspond to positive (negative) effects of previous state on current state. In the scenario with no heterogeneity, a negligible value of HPDH (0.01) was used to avoid numerical errors when simulating random effects from a bivariate normal distribution (Equation 3) and when computing the relative bias.

### 4.2 Model fitting

To keep the problem tractable, mean survival was kept constant throughout life in simulations. Furthermore, we only analyzed breeding trajectories: Observed reproductive life span was treated as data. In other words, although the true data‐generating mechanism is a joint model of breeding success and survival, only data on breeding success were analyzed with probabilistic models (Table 2). Models were fitted with software R v.3.2.3 (R Development Core Team, 2015) using the function *glmer* from the library *lme4* (Bates, Maechler, Bolker, & Walker, 2013) on a HP Compaq LA2306x desktop (Intel (R), Xeon (R) CPU E5‐2630, 2.30 GHZ, 32 Go RAM). We specified 5 quadrature points for the adaptive Gauss–Hermite approximation to the log‐likelihood for accurate random effect estimation (Lesaffre & Spiessens, 2001).

**Table 2:** Models for analyzing breeding trajectories. $\alpha_{i}^{*}$ is an individual univariate random effect because only breeding success was analyzed at this stage

$M_{\mathrm{nil}}$	${\text{success}}_{it}∼\text{Bernoulli}\left({\text{logit}}^{-1}\left(\mu \right)\right)$
$M_{\mathrm{NTLH}}$	${\text{success}}_{it}∼\text{Bernoulli}\left({\text{logit}}^{-1}\left(\mu +\gamma \times {\text{success}}_{i(t-1)}\right)\right)$
$M_{\mathrm{HPDH}}$	${\text{success}}_{it}∼\text{Bernoulli}\left({\text{logit}}^{-1}\left(\mu +\alpha_{i}^{*}\right)\right)$
$M_{\mathrm{full}}$	${\text{success}}_{it}∼\text{Bernoulli}\left({\text{logit}}^{-1}\left(\mu +\gamma \times {\text{success}}_{i(t-1)}+\alpha_{i}^{*}\right)\right)$

### 4.3 Inference

Our aims were to assess the empirical validity of the current NTLH framework to draw inferences about the processes generating variation in life histories. For each fitted model, estimated parameters were used to predict individual LRS conditional on the observed survival trajectory. To quantify the discrepancy between the predicted and observed distribution of LRS, Kolmogorov–Smirnov tests have been used in previous studies of NTLH, but were found underpowered (Bonnet & Postma, 2016). We used the Earth Mover Distance to compare two histograms or distributions. Each histogram may be viewed as a pile of sand and the Earth Mover Distance reflects the amount of sand multiplied by the distance needed to turn one pile into the other (Gottschlich & Schuhmacher, 2014). A smaller Earth Mover Distance reflects a better match between predictions and observations.

For each dataset and fitted model, estimated parameters $\left(\hat{\mu },\hat{\gamma },{\hat{\sigma }}_{\mathrm{repro}}\right)$ were stored to assess bias and to compute individual stochasticity and the scaled entropy. Entropy is a measure of randomness in transitions between breeding success and failure (Tuljapurkar et al., 2009). The scaled entropy varies between 0 and 1, with 1 corresponding to complete randomness in the succession of states in the trajectory. It has been argued that HPDH should decrease entropy (Bonnet & Postma, 2016; Jenouvrier et al., 2015; Tuljapurkar et al., 2009) and that entropy could thus be used to infer the presence of HPDH. We used estimated parameters $\left(\hat{\mu },\hat{\gamma }\right)$ to compute the within‐individual variance ${\hat{\sigma }}_{\mathrm{within}}^{2}$for the breeding trajectory 01. Since variances are additive, the total variance is the sum the rightmost terms in Equation 11. The estimated within‐individual variance term was compared to its true value for each simulation scenario to assess bias, that is whether $\sigma_{\mathrm{within}}^{2}={\hat{\sigma }}_{\mathrm{within}}^{2}$ or not.

Finally, the log‐likelihood of each simulated dataset under each model (Table 2) was recorded to compute the Bayesian information criteria (BIC; Link & Barker, 2009). BIC weights $\left({\hat{\omega }}_{\mathrm{BIC}}\right)$ were then calculated assuming that random individual effects count for 1 additional (variance) parameter. We used BIC rather than Akaike information criterion (AIC) because the later tends to favor overcomplex models in large data sets (chapter 7 in Link & Barker, 2009). Results were qualitatively similar with AIC (Appendix S3).

For legibility, only results for scenarios where there was no individual‐level correlation in HPDH *(*cor = 0) are presented below. These results are qualitatively the same for other scenarios (Appendix S3).

## 5 Results

**Figure 1:**
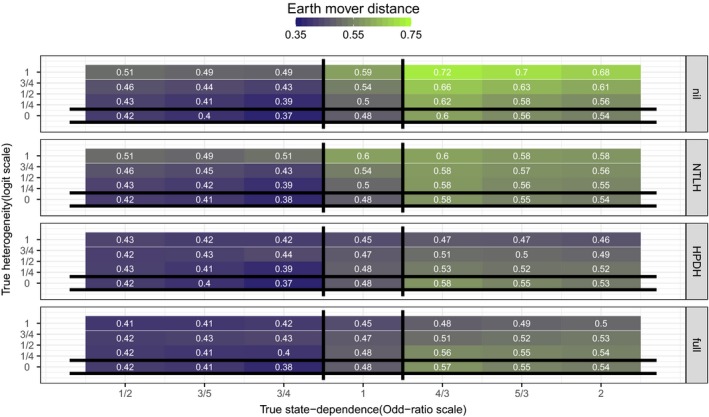
Tile‐plots of the average estimated Earth Mover Distance (across 500 simulated datasets) between the observed and predicted distribution of LRS for each simulation scenario. True values of state dependence (*e* ^*γ*^ on the Odds‐Ratio scale) and HPDH (*σ*_repro_) are on the *x*− and *y*− axes, respectively. Each panel corresponds to one of the four models used to analyze data. Vertical black lines bracket scenarios in which heterogeneity ($M_{\mathrm{HPDH}}$) is the true data‐generating mechanism. Horizontal black lines bracket scenarios in which state dependence ($M_{\mathrm{NTLH}}$) is the true data‐generating mechanism. At the intersection, $M_{\mathrm{nil}}$ is the true data‐generating mechanism. Everywhere else, $M_{\mathrm{full}}$ is the true data‐generating mechanism. The true data‐generating model should have the smallest Earth Mover Distance. Actual values (rounded to the nearest integer) are displayed on each tile

Figure 1 summarizes results with the Earth Mover Distance: Irrespective of the true data‐generating mechanism, all fitted models could predict the observed population‐level distribution of LRS in a similar manner. Exceptions were for models $M_{\mathrm{nil}}$ and $M_{\mathrm{NTLH}}$ (that is models excluding HPDH) whose Earth Mover Distance was the greatest in scenarios with small to large true HPDH. This distance was further increased with increased value of true positive state dependence (*e* ^*γ*^ > 1) and increased value of the individual‐level correlation in HPDH (Appendix S3: Fig. S1). In scenarios with both state dependence and HPDH, model $M_{\mathrm{HPDH}}$ had the smallest distance, even though it was not the true data‐generating mechanism. In other words, a misspecified model could outperform the true model at predicting a LRS distribution that was most similar to the observed one.

**Figure 2:**
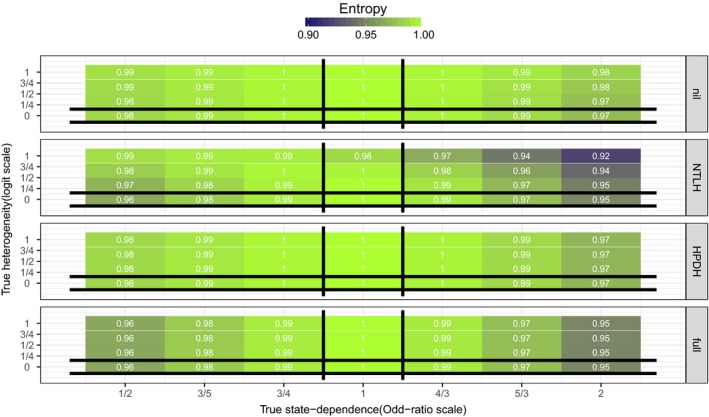
Tile‐plots of the average estimated scaled entropy (across 500 simulated datasets) in each simulation scenario. True values of state dependence (*e* ^*γ*^ on the Odds‐Ratio scale) and HPDH (*σ*_repro_) are on the *x*− and *y*− axes, respectively. Each panel corresponds to one of the four models used to analyze data. Vertical black lines bracket scenarios in which $M_{\mathrm{HPDH}}$ is the true data‐generating mechanism. Horizontal black lines bracket scenarios in which $M_{\mathrm{NTLH}}$ is the true data‐generating mechanism. At the intersection, $M_{\mathrm{nil}}$ is the true data‐generating mechanism. Everywhere else, $M_{\mathrm{full}}$ is the true data‐generating mechanism. Actual values (rounded to the nearest integer) are displayed on each tile

Figure 2 summarizes results with respect to scaled entropy: Irrespective of the true data‐generating mechanism, parameter estimates from the different fitted models could result in a similar scaled entropy. Exceptions were for model $M_{\mathrm{NTLH}}$, for which the computed scaled entropy was the smallest in scenarios with both large true HPDH and large, positive, true positive state dependence (*e* ^*γ*^ > 1). Scaled entropy further decreased with increased value of the individual‐level correlation in HPDH (Appendix S3: Fig. S2). This behavior was also apparent for models $M_{\mathrm{HPDH}}$ and $M_{\mathrm{full}}$ although the ranking was invariable with the smallest, intermediate, and largest scaled entropy for $M_{\mathrm{NTLH}}$ *,* $M_{\mathrm{full}}$ and $M_{\mathrm{HPDH}}$, respectively. All models, whether they included state dependence, heterogeneity, or excluded them both, could generate similar values of scaled entropy: Scaled entropy was insensitive to the true data‐generating mechanism (Appendix S3: Fig. S2).

**Figure 3:**
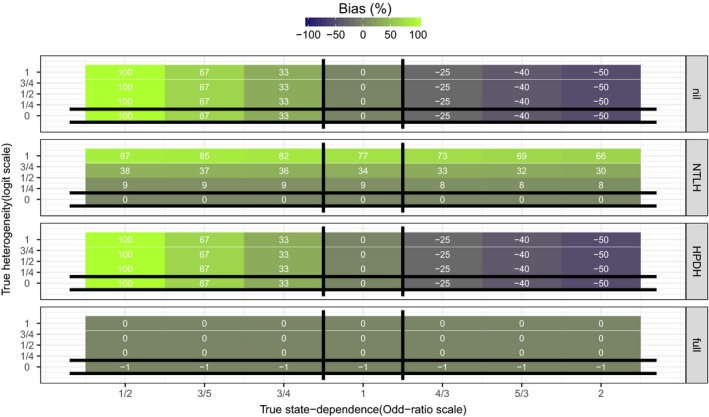
Tile‐plots of the average bias in estimated state dependence $\hat{\gamma }$ (across 500 simulated datasets) for each simulation scenario. True values of state dependence (*e* ^*γ*^ on the Odds‐Ratio scale) and HPDH (*σ*_repro_) are on the *x*− and *y*− axes, respectively. Each panel corresponds to one of the four models used to analyze data. Vertical black lines bracket scenarios in which $M_{\mathrm{HPDH}}$ is the true data‐generating mechanism. Because $M_{\mathrm{HPDH}}$ excludes *γ*, estimates ($\hat{\gamma }$) are by definition exactly 0. Horizontal black lines bracket scenarios in which $M_{\mathrm{NTLH}}$ is the true data‐generating mechanism. At the intersection, $M_{\mathrm{nil}}$ is the true data‐generating mechanism. Everywhere else, $M_{\mathrm{full}}$ is the true data‐generating mechanism. Estimates from the true data‐generating model should have no bias on average. Actual bias values (rounded to the nearest integer) are displayed on each tile. Biases larger than 100% in magnitude were capped at 100%

Figure 3 shows the bias in estimated state dependence $\left(\hat{\gamma }\right)$. When data were analyzed with a correctly specified model, state dependence estimates were on average unbiased. However, when data were analyzed with a different model than the true data‐generating mechanism, estimates from all models other than $M_{\mathrm{full}}$ were biased. In particular, estimates from model $M_{\mathrm{NTLH}}$ were always positively biased when HPDH was present in the data (Appendix S3: Fig. S3). Even in scenarios where true state dependence was nil (*e* ^*γ*^ = 1), estimates from model $M_{\mathrm{NTLH}}$ were positively biased, with the magnitude of the bias depending only on the magnitude of true HPDH (Appendix S3: Fig. S3).

**Figure 4:**
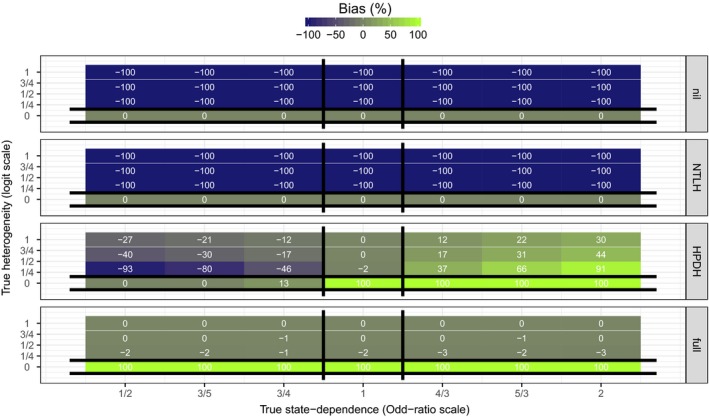
Tile‐plots of the average bias in estimated HPDH ${\hat{\sigma }}_{\mathrm{repro}}$ (across 500 simulated datasets) for each simulation scenario. True values of state dependence (*e* ^*γ*^ on the Odds‐Ratio scale) and HPDH (*σ*_repro_) are on the *x*− and *y*− axes, respectively. Each panel corresponds to one of the four models used to analyze data. Vertical black lines bracket scenarios in which $M_{\mathrm{HPDH}}$ is the true data‐generating mechanism. Horizontal black lines bracket scenarios in which $M_{\mathrm{NTLH}}$ is the true data‐generating mechanism. Because $M_{\mathrm{NTLH}}$ excludes *σ*_repro_, estimates (${\hat{\sigma }}_{\mathrm{repro}}$) are by definition exactly 0. At the intersection, $M_{\mathrm{nil}}$ is the true data‐generating mechanism. Everywhere else, $M_{\mathrm{full}}$ is the true data‐generating mechanism. Estimates from the true data‐generating model should have no bias on average. Actual bias values (rounded to the nearest integer) are displayed on each tile. Biases larger than 100% in magnitude were capped at 100%

Figure 4 shows the bias in estimated HPDH ${\hat{\sigma }}_{\mathrm{repro}}$. When data were analyzed with a correctly specified model, HPDH estimates were on average unbiased. However, when data were analyzed with a different model than the true data‐generating mechanism, estimates were in general biased. In particular, estimates from model $M_{\mathrm{HPDH}}$ were always biased when data were generated with true state dependence (Appendix S3: Fig. S4). In scenarios where true HPDH was nil ($\sigma_{\mathrm{repro}}=0$), estimates from model $M_{\mathrm{HPDH}}$ or $M_{\mathrm{full}}$ were positively biased. In scenarios where both state dependence and HPDH were truly present, estimates from model $M_{\mathrm{HPDH}}$ were always biased with the sign and severity of the bias depending on the sign and magnitude of true state dependence (Appendix S3: Fig. S4).

**Figure 5:**
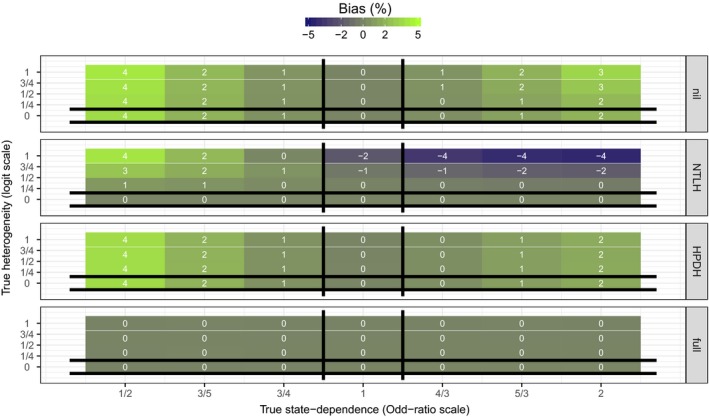
Tile‐plots of the average bias in estimated within‐individual variance (${\hat{\sigma }}_{\mathrm{within}}^{2}$) for a 01 trajectory in breeding success of an average individual for each simulation scenario. True values of state dependence (*e* ^*γ*^ on the Odds‐Ratio scale) and HPDH (*σ*_repro_) are on the *x*− and *y*− axes respectively. Each panel corresponds to one of the four models used to analyze data. Vertical black lines bracket scenarios in which $M_{\mathrm{HPDH}}$ is the true data‐generating mechanism. Horizontal black lines bracket scenarios in which $M_{\mathrm{NTLH}}$ is the true data‐generating mechanism. At the intersection, $M_{\mathrm{nil}}$ is the true data‐generating mechanism. Everywhere else, $M_{\mathrm{full}}$ is the true data‐generating mechanism. Estimates from the true data‐generating model should have no bias on average. Since variances are additive, any bias can blow up with the large sample considered in our simulations of a panel of 1*,*000 individuals. Actual bias values (rounded to the nearest integer) are displayed on each tile

Results with respect to within‐individual variance estimation, or individual stochasticity sensu Caswell (2009), are summarized in Figure 5. When data were analyzed with a correctly specified model, individual stochasticity estimates were on average unbiased. The only exception was in scenarios with both HPDH and state dependence: Estimates from model $M_{\mathrm{full}}$ were slightly biased with the bias depending on the individual‐level correlation cor (Appendix S3: Fig. S5). When data were analyzed with a different model than the true data‐generating mechanism, estimates were in general biased. In particular, estimates from model $M_{\mathrm{NTLH}}$ were always biased when HPDH was present in the data. Likewise, estimates from model $M_{\mathrm{HPDH}}$ were biased when state dependence was present in the data.

**Figure 6:**
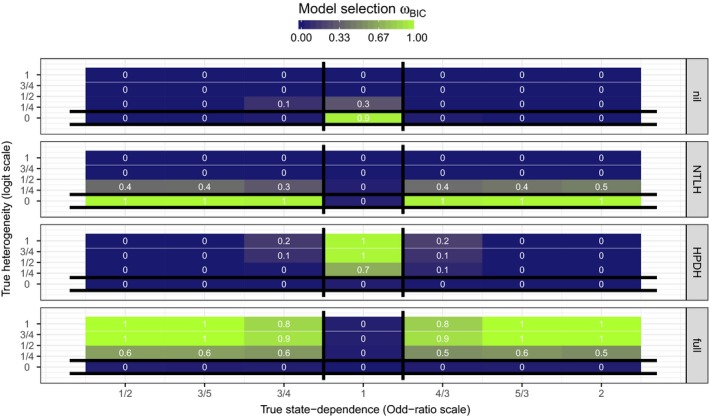
Tile‐plots of the mean estimated $\left({\hat{\omega }}_{\mathrm{BIC}}\right)$ (across 500 simulated datasets) for each model and each simulation scenario. True values of state dependence (*e* ^*γ*^ on the Odds‐Ratio scale) and HPDH (*σ*_repro_) are on the *x*− and *y*− axes, respectively. Each panel corresponds to one of the four models used to analyze data. Vertical black lines bracket scenarios in which $M_{\mathrm{HPDH}}$ is the true data‐generating mechanism. Horizontal black lines bracket scenarios in which $M_{\mathrm{NTLH}}$ is the true data‐generating mechanism. At the intersection, $M_{\mathrm{nil}}$ is the true data‐generating mechanism. Everywhere else, $M_{\mathrm{full}}$ is the true data‐generating mechanism. The best predictive model, which would be selected for inference, has the largest weight. Actual values (rounded to one decimal) are displayed on each tile

Inference with BIC is summarized in Figure 6. Across all the scenarios, the information theoretic approach was able to identify the correct data‐generating mechanism among the competing models. Although a small amount of HPDH or state dependence were more difficult to detect with certainty, ${\hat{\omega }}_{\mathrm{BIC}}$ were always the largest for the true data‐generating mechanism: Using BIC in a multimodel framework avoided the problems linked to model misspecification detailed in Figures 1, 2, 3, 4, 5 above (see also Appendix S3: Figs S6 and S7).

## 6 Discussion

### 6.1 Inference with misspecified models

An important methodological choice in evolutionary ecology is that of an appropriate null model (Pigliucci & Kaplan, 2006; chapter 10). With simulations, we investigated whether the observed distribution of LRS could reflect unambiguously the action of state dependence, HPDH, or both on life‐history trajectories. Using the Earth Mover Distance, we found that the true data‐generating mechanism was not necessarily the one that predicted best LRS. LRS predictions from a model including only state dependence or HPDH could be closer to the observed distribution of LRS, even though the true data‐generating mechanism involved both state dependence and HPDH. The ability of statistical models to predict well the population‐level distribution of LRS tells little about the true data‐generating mechanism at the individual level. Excellent predictive ability may result from biased estimates (Shmuéli, 2010), which may have plagued previous studies. Biased estimates do not allow accurate inferences on whether variability across individual life histories is generated by chance alone or not. That is, they do not allow inferring whether there are unobserved individual features on which natural selection may act provided that the necessary conditions are met (Fox, Roff, & Fairbairn, 2001). With that goal in mind, a model's ability to predict the observed distribution of LRS is not sufficient; the selected model has to provide more than population‐level predictions, it has to be the one that reflects best the biological processes that gave rise to the data at the individual level (Cam et al., 2016).

We investigated the use of scaled entropy as a summary statistic for inference about the processes generating variation in life‐history trajectories. Entropy is a measure of randomness in transitions between states in a sequence: It measures uncertainty in predicting the next state (Adami, 2016). State dependence by definition (for a first order Markovian process) assumes that the realization of the random variable “breeding success” in year *t* + 1 is predictable from knowledge of state at *t*. Hence, a decrease in scaled entropy is expected if estimated state dependence is non‐nil ($\hat{\gamma }\neq 0$). Consequently, scaled entropy was smallest for $M_{\mathrm{NTLH}}$ when the bias in ($\hat{\gamma }$) was largest. In populations with HPDH, important individual‐level covariates are assumed to influence transitions among states, but these covariates are unobserved. Their effect is subsumed into an individual‐level random effect. As a result, transitions are difficult to predict for a randomly chosen individual in the population, since important information on this individual is missing to begin with. Consequently, the scaled entropy computed from parameters estimated with a model with only HPDH was always the largest. Finally, scaled entropy was intermediate for the model incorporating both state dependence and HPDH. Across the different scenarios, parameter estimates from this model were unbiased and allowed accurate computation of the scaled entropy. The latter cannot be used for inference about the true data‐generating process because its computation is conditional on the model used being correctly specified: Entropy cannot tell whether a model provides a good fit or not to a given dataset.

Model misspecification seriously limits the usefulness of a null model in life‐history studies: Because parameter estimates can be biased, testing whether they are nil or not is moot. Besides, there are statistical hurdles involved in the direct comparison of $M_{\mathrm{NTLH}}$, $M_{\mathrm{HPDH}}$, and $M_{\mathrm{full}}$. Both $M_{\mathrm{HPDH}}$ and $M_{\mathrm{NTLH}}$ are simpler versions of $M_{\mathrm{full}}$, but the direct comparison of $M_{\mathrm{NTLH}}$, which is a generalized linear model, and of $M_{\mathrm{HPDH}}$, which is a generalized linear mixed model, can be involved (Appendix S4). Our results showed that standard information theoretic tools such as ${\hat{\omega }}_{\mathrm{BIC}}$ (Link & Barker, 2009) or ${\hat{\omega }}_{\mathrm{AIC}}$ (Burnham & Anderson, 2002; Burnham & White, 2002) could be used to identify a model specification that best approximated the correct data‐generating mechanism. Our simulations were carried out with both a large number of individuals and a large reproductive life span in order to allow asymptotic justifications to hold. In practice, sample size may be small and asymptotic justifications may not hold. To achieve correct model specification, it is paramount to consider a set of candidate models simultaneously to assess their fit, and not to rely on LRS prediction or entropy computation for inferring the true data‐generating mechanism driving the intra‐ and interindividual dynamics of change among states in life‐history trajectories.

### 6.2 Testing demographic heterogeneity in practice

State dependence and HPDH can both drive the dynamics of change among states in individual life histories. When both processes were operating, models ignoring state dependence resulted in overestimating HPDH. Likewise, ignoring HPDH resulted in overestimating state dependence, a result unanticipated by previous methods used to test NTLH. This statistical bias occurred because past state is a random variable: Treating it as fixed when put in the right‐hand side of Equation 2 introduces endogeneity (Hsiao, 2014). Endogeneity is a special case of omitted variable bias, in which the bias arises because one of the variables used to explain the other is itself caused by the phenomenon it seeks to explain. Because it can itself be the result of unobserved HPDH, past state acts as a proxy for “individual quality”, resulting in spurious state dependence (Hsiao, 2014). As a result of spurious state dependence, individual stochasticity sensu Caswell (2009) was underestimated when data with true HPDH were analyzed with a model including only state dependence (Figure 5). When data with both true HPDH and true state dependence are analyzed with the misspecified model $M_{\mathrm{NTLH}}$, estimated state dependence is exaggerated and individual stochasticity underestimated (when true state dependence is positive) or overestimated (when true state dependence is negative). In other words, the current NTLH framework may lead to infer too much or too little stochastic variability in life histories (i.e., individual stochasticity sensu Caswell, 2009) than there really is.

We focused on the variance in states along a trajectory to define both a within‐ and between‐individual component, which is consistent with previous investigations focusing on HPDH (Cam et al., 2002; Chambert et al., 2014). In contrast, previous studies of the NTLH defined a within‐ and a between‐trajectory variance, which is only accessible via simulations (Steiner et al., 2010). These simulations emulate variations due to death of individuals sharing the same trajectory, the latter being an assumption since it is defined a priori, independently of time of death. In fact, it is impossible to know whether any two individuals are truly sharing the same trajectory. Moreover, simulations depend on the model which was used to estimate the required parameters. Importantly, these simulations assume that the model specification is correct and cannot diagnose any statistical bias since there is no model‐independent measure of the within‐ and between‐trajectory variances. Any bias in estimated parameters will necessarily trickle down in simulations that are conditional on unknown parameters estimated from empirical data. It is paramount to address the potential problem of model misspecification, and the resulting biased estimates, if reliable inferences on life‐history evolution are to be drawn from such simulations.

Because of estimation biases, the neutral model fits many longitudinal life‐history datasets and mirrors the situation observed in community ecology, where neutral models were found to successfully predict species abundance distributions (SAD). It was later shown that predicting SAD did not provide robust support for the Neutral Theory of Biodiversity (Chave, 2008): Both neutral and non‐neutral models can predict the same pattern (Chave, Muller‐Landau, & Levin, 2002). Using data on American college basket‐ball competition recasted as SAD, Warren II et al. (2011) showed that neutral models of community ecology could very well predict the patterns in these data: A non‐neutral process at a microlevel can generate a seemingly neutral pattern at a macrolevel. Similarly, we demonstrate here that in the current NTLH framework, predicting LRS distributions does not provide robust evidence of neutrality.

### 6.3 Old wine in new bottles

The problem of biased estimates has been diagnosed more than 30 years ago by econometricians (Heckman, 1981): “misspecification of the heterogeneity process gives rise to an erroneous estimate of the impact of the true effect of past state on current outcome.” Econometricians now always consider a model with both state dependence and unobserved heterogeneity (e.g., Arulampalam, Booth, & Taylor, 2000; Bartels, Box‐Steffensmeier, Smidt, & Smith, 2011; Halliday, 2008; Hsiao, 2014) to avoid mis‐estimating either. In scenarios where true state dependence *γ* was negative, consistent with a trade‐off between current and future reproduction, estimated state dependence $\hat{\gamma }$ could be positive. The bias was always positive and resulted from model misspecification when HPDH was in fact present in the data, but ignored in the analysis. This systematic positive bias may have contributed to the elusiveness of trade‐offs in empirical studies: Overestimating the magnitude (Type‐M error) of state dependence can further lead to a sign error (Type‐S error; Gelman & Tuerlinckx, 2000). To ignore HPDH, a priori can be detrimental to the study of trade‐offs in wild populations.

Negative state dependence is classically interpreted as a cost of reproduction, which can hardly be considered a neutral process with respect to natural selection (Flatt & Heyland, 2011). As Munoz and Huneman (2016) underscored in the context of the Neutral Theory of Biodiversity, “one can have neutral patterns with non‐neutral processes”. The current formulation of NTLH may obfuscate the difference between patterns and processes. While the realization of stochastic processes governed by state dependence creates variation among individual trajectories that may be evolutionary neutral, the biological and evolutionary processes at the origin of state dependence need not themselves be neutral. Consequently, it is necessary to clarify why the state dependence model sensu (Heckman, 1981), which also underpins population projection matrix models (Caswell, 2001; Lefkovitch, 1965), is an evolutionary neutral model of life‐history evolution. NTLH requires a philosophical clarification like the one Munoz and Huneman (2016) recently provided for the Neutral Theory of Biodiversity. Although beyond the scope of this study, we offer some thoughts below, focusing on what neutrality means in life‐history studies.

### 6.4 Neutrality in Life‐history Studies

Trade‐offs between life‐history traits are commonly understood as allocation constraints acting on the development and physiology of organisms. They are a cornerstone of evolutionary biology (Roff, Mostowy, & Fairbairn, 2002) and translate into negative state dependence in statistical models (Nichols, Hines, Pollock, Hinz, & Link, 1994). Evidencing trade‐offs in natural populations has proven a difficult endeavor in spite of clear and straightforward theoretical expectations about their existence (Metcalf, 2016; Morano, Stewart, Sedinger, Nicolai, & Vavra, 2013; Reznick et al., 2000). In evolutionary biology, few researchers would equate a trade‐off between current reproduction or future survival as evidence of neutrality. Yet NTLH holds that neutrality in life histories stems from state dependence that is the effect of previous state on current state. In fact, in its current formulation, NTLH provides a model that is more parsimonious than neutral (Munoz & Huneman, 2016): Parsimony is enacted by the nullification of the heterogeneity (variance) parameter. However, it is unclear why it is more appropriate to nullify this parameter, rather than the state dependence parameter in the first place. HPDH affects in no small way the interpretation of logistic regression output, and ignoring it a priori is not recommended (Mood, 2010). Moreover, NTLH is agnostic to the sign of state dependence: There is no specific prediction about whether positive or negative state dependence should be expected in a given context, nor whether its magnitude can fluctuate. Trade‐offs are expected to be expressed most acutely when conditions are harsh or competition is strong (van Noordwijk & de Jong, 1986) and should be considered context‐dependent rather than static (Roff et al., 2002).

An analogy to two other disciplines, namely population genetics and community ecology, was called upon to promote consideration of nonselective mechanisms in life‐history studies (Steiner & Tuljapurkar, 2012). The underlying motivation was to avoid an automatic presumption of adaptationism (Gould & Lewontin, 1979) that could flourish under the label of “individual quality” (Bergeron et al., 2011; Steiner & Tuljapurkar, 2012). As a result, NTLH was developed to provide a baseline model that could be used as a working null hypothesis for empiricists (Steiner & Tuljapurkar, 2012). However, in contrast to population genetics or community ecology (Chave, 2008; Leigh, 2007), NTLH has not been seized upon by empiricists. This state of affairs calls for an in‐depth investigation into the limits of drawing analogies between different disciplines developing neutral theories and using null models.

In NLTH, neutrality is deduced not because there are no biological differences between individuals, but because these differences are deemed fitness‐irrelevant. The latter conclusion is reached when a state dependence statistical model can predict well the population‐level distribution of LRS without assuming between‐individual differences (heterogeneity) in vital rates beyond the usual differences in age‐ and stage‐structured populations. LRS plays the same role as allele frequencies in population genetics, or SAD in community ecology. In the latter two disciplines, the emphasis is on accounting for temporal changes in the population‐level distribution of allele frequencies and community‐level SAD (Chave, 2008), respectively. In both population genetics and community ecology, a microlevel process stemming from the finite size of populations, drift, can induce random change in the distribution of a macrolevel statistic (allele frequency or SAD). In NTLH, state dependence provides a mechanism to explain change in states within an individual life‐history, but does not necessarily explain change in the population‐level LRS distribution over time. Support for NTLH was claimed from predicting accurately the LRS distribution at a specific time point, not from accounting for a change over time in that distribution. While change in the magnitude or sign of state dependence can generate different LRS distribution (Appendix S2), the current emphasis of NTLH is not on the origin, the sign, or the magnitude of the state dependence parameter. These blind spots call for further theoretical elaborations of NTLH and renewed attention on the focus of tests of NTLH: predicting from individual/microlevel processes (i) the population/macrolevel LRS distribution at a specific time point, or (ii) changes in that distribution over time.

## 7 Conclusion

Researchers interested in the evolutionary significance of interindividual variations in longitudinal trajectories should not use LRS or entropy to infer the correct data‐generating mechanism behind life histories. Neither LRS prediction nor entropy estimation can diagnose model misspecification. NTLH studies (e.g., Tuljapurkar et al., 2009) should be re‐evaluated with standard inferential tools, such as information criteria. In theory, the latter can be used to compare and accurately select a model accounting for the data‐generating mechanisms behind longitudinal life‐history data. Although with real data collected on wild populations the true data‐generating mechanism is, in general, unknown and out of reach (Burnham & Anderson, 2004; Link & Barker, 2009), investigators should start from a set of statistical models reflecting a complete set of nonmutually exclusive hypotheses (including alternative biological and evolutionary scenarios) on individual life‐history evolution (Browne et al., 2007; Cam et al., 2013; Chambert, Rotella, & Higgs, 2014; 2013). Then, they should proceed with a multihypothesis framework (Chamberlin, 1965) based on information theoretic inferential tools (BIC, AIC, or analogs in the Bayesian framework; Gelman, Hwang, & Vehtari, 2014; Link & Barker, 2009). These criteria allow determining a model whose complexity (e.g., non‐Gaussian HPDH, second‐order Markovian state dependence) is supported by the data at hand (Burnham & Anderson, 2004; Link & Barker, 2009), comparing non‐nested models and assessing the relative importance of heterogeneity and state dependence in individual life‐history evolution.

## Conflict of Interest

None declared.

## Authors’ contribution

All the authors have equally contributed to this work. MA, LMA, and EC involved in conceptualization, analysis, and writing.

## Data Accessibility

Data available from the Dryad Digital Repository: http://dx.doi.org/10.5061/dryad.874p9
