# Supplementary material for: Wolf in sheep's clothing: Model misspecification undermines tests of the neutral theory for life histories
Source: Ecol Evol. 2017 Apr 4;7(10):3348–61. doi: 10.1002/ece3.2874 (PMC5433986; doi:10.1002/ece3.2874)
Supplement: Supplementary file 1 [file ECE3-7-3348-s001.docx]

## Appendix S1: Citation Analysis

We generated a citation report from a literature search conducted in *Web of Science* in August 2015 using the following keywords: "Heterogeneity life histories" OR "Heterogeneity vital rates" OR "Neutral theory life histories" OR "Dynamic heterogeneity" OR "Fixed heterogeneity" OR "Individual heterogeneity" OR "Unobserved heterogeneity" OR "Frailty" OR "Latent fitness" OR "Latent heterogeneity" OR "Individual stochasticity" (see Cam et al. 2016 for a thorough discussion of the terminology); with "OR" indicating that if any of these terms were found within the general topic of a citation, the citation would then be selected by the search engine. The keywords had to be part of the general "topic" rather than the "title" of the selected publications (too restrictive). The search was further restricted to the fields of "environmental science and ecology", "evolutionary biology", and "zoology" within the "Science technology" domain (excluding "social science" and "arts humanities" domains). To weed-out irrelevant articles and reviews, we further excluded scientific journals that did not fall within the realm of ecological and evolutionary studies (*e.g.* ecological economics). The search was performed to include all peer-reviewed ’articles’ and ’reviews’ published between the 1st of January 1990 and the 31st of December 2014. From the literature search, we selected 157 publications cited a total of 3*,*644 times (23*.*21 average citation per item). We report both the number of articles and reviews published each year, as well as the associated number of citation for the selected publications from the 1st of January 1990 up to the 31st of December 2014 (source: *Web of Science*).


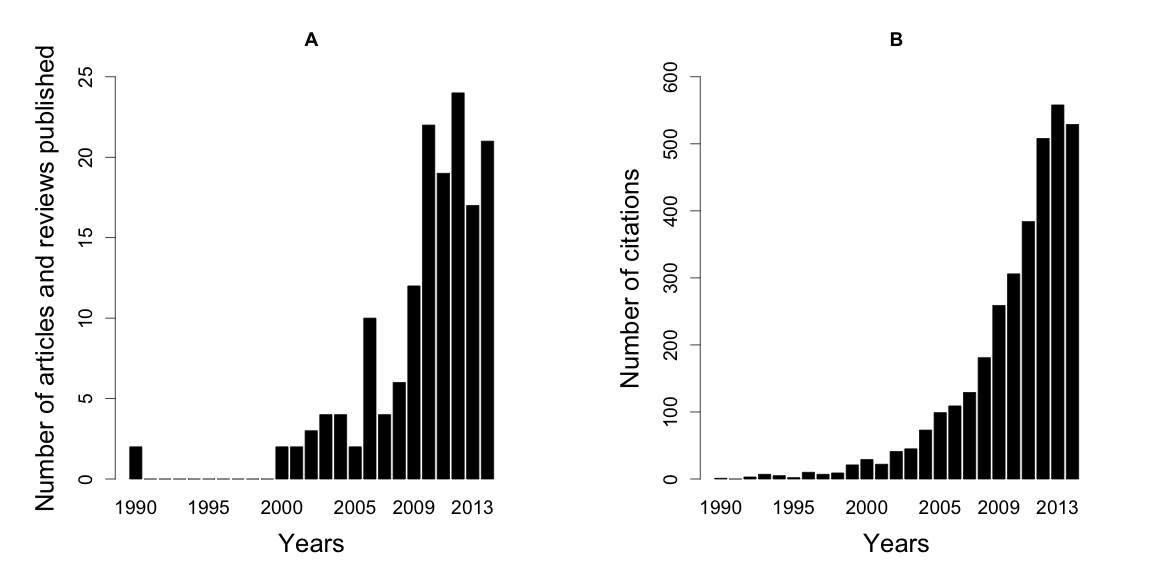


Figure S1: Citation report of ecological and evolutionary studies of heterogeneity in life histories. We generated a citation report from a literature search conducted in *Web of Science* in August 2015. The search was performed to include all peer-reviewed ’articles’ and ’reviews’ published between the 1st of January 1990 and the 31st of December 2014. From the literature search, we selected 157 publications cited a total of 3*,*644 times (≈ 23 average citations per item). We report both the number of articles and reviews published each year (left panel), as well as the associated number of citations for the selected publications from the 1st of January 1990 up to the 31st of December 2014 (right panel).
