# Supplementary material for: Wolf in sheep's clothing: Model misspecification undermines tests of the neutral theory for life histories
Source: Ecol Evol. 2017 Apr 4;7(10):3348–61. doi: 10.1002/ece3.2874 (PMC5433986; doi:10.1002/ece3.2874)
Supplement: Supplementary file 2 [file ECE3-7-3348-s002.docx]

## Appendix S2: Lifetime Reproductive Success Simulations


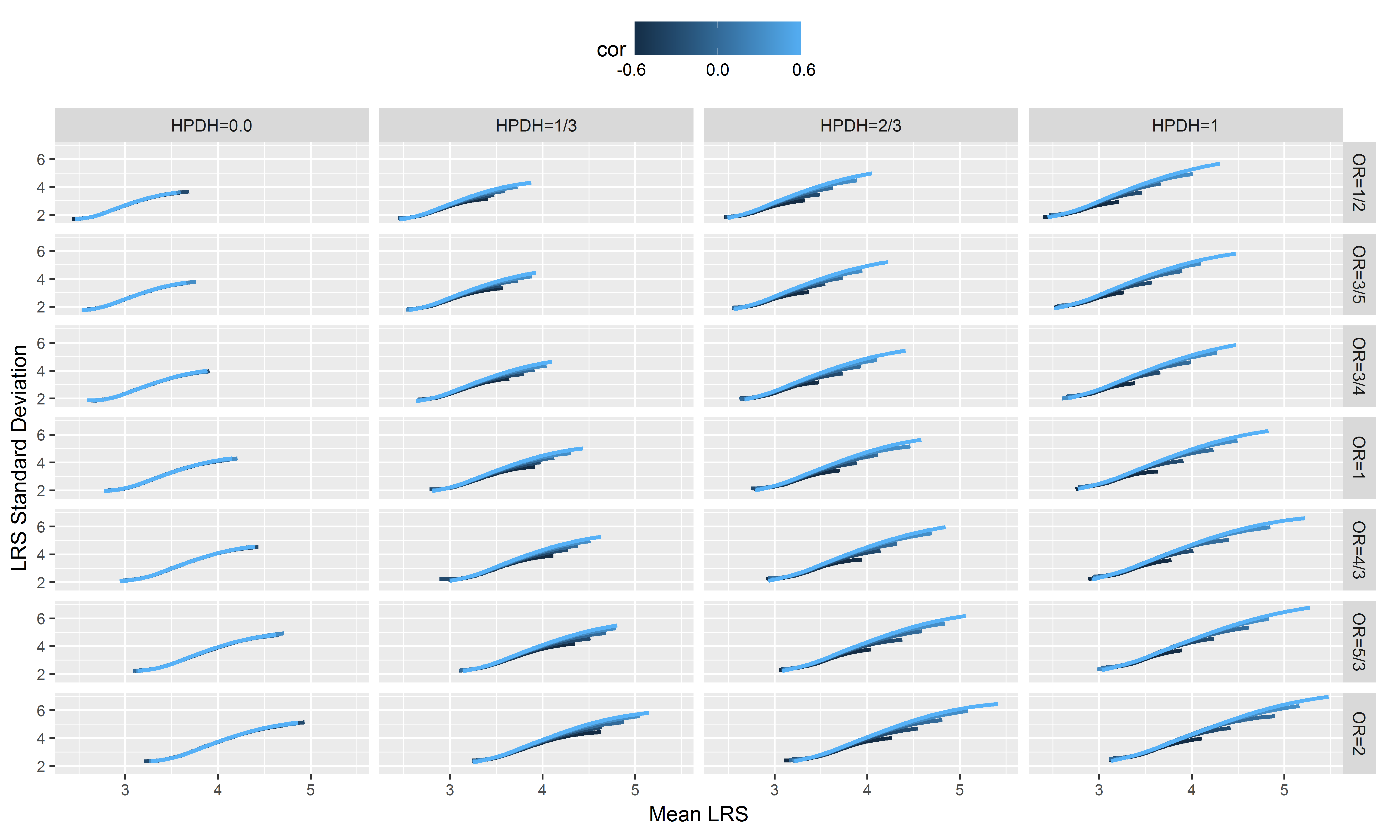


Figure S1: smoothed curves of the standard deviation of (dispersion) *versus* mean LRS across the different scenarios. The leftmost column corresponds to no HPDH, and the middle row to no state-dependence. *OR* stands for the Odds-Ratio, that is $e^{\gamma}$. For the scenario with no HPDH or state-dependence (leftmost column, middle row): the relationship is positive and is insensitive to the value of cor. The middle row corresponds to HPDH only: the effect of HPDH is to increase both the mean and the dispersion of LRS, although this is moderated by the value of $\mathrm{cor}$. When there is a trade-off between survival and breeding success ($cor<0$), both the mean and dispersion of LRS were smaller compared to the case with no heterogeneity at all. However, when there is positive covariation between survival and breeding success ($cor>0$), both the mean and dispersion of LRS were larger. The leftmost column corresponds to state-dependence only: the effect of state-dependence is also to increase both the mean and the dispersion of LRS. When both state-dependence and HPDH were present in the data-generating mechanism, there was an increase in both the mean and the dispersion of LRS, although this was moderated by the sign and value of $\mathrm{cor}$.


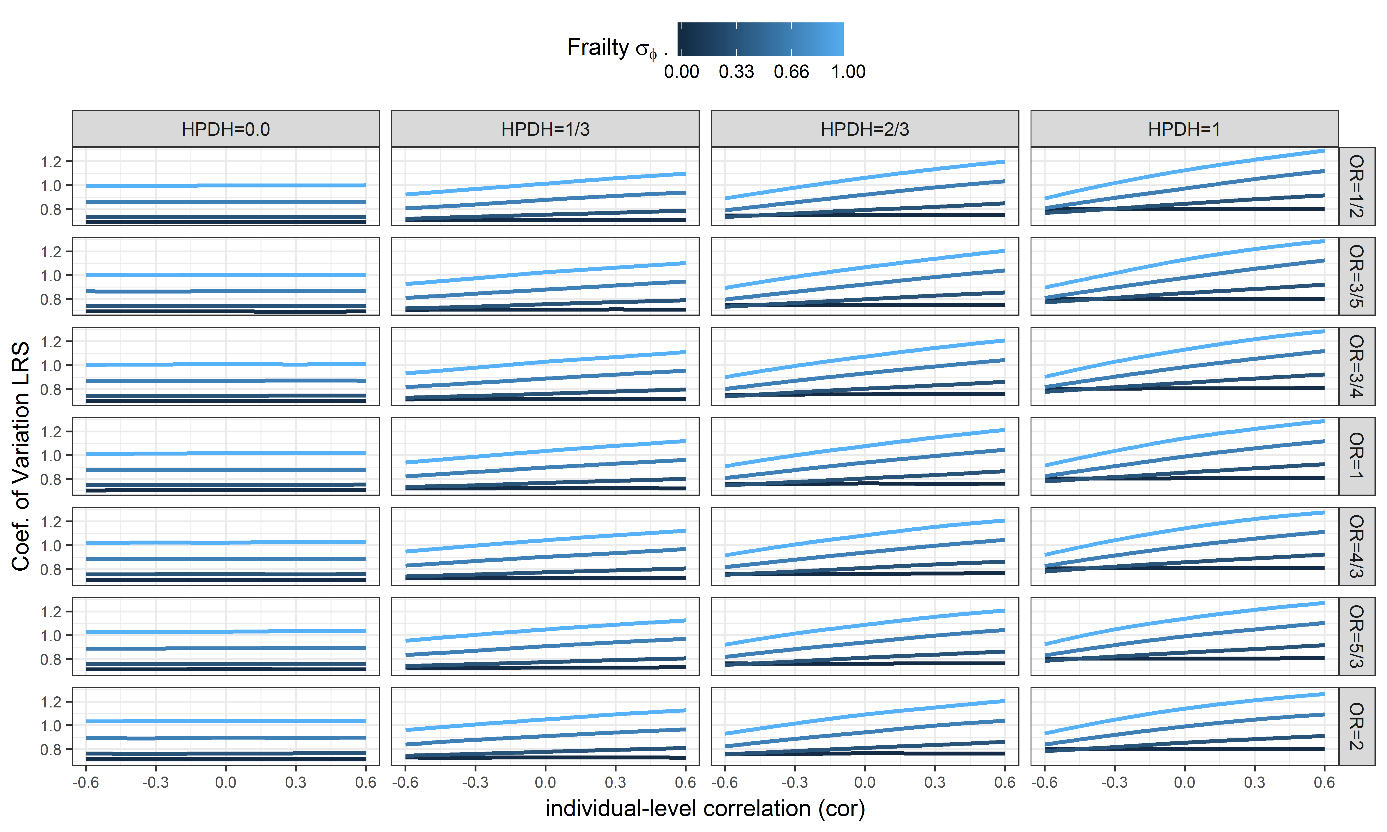


Figure S2: smoothed curves of the coefficient of variation of population-level LRS *versus* the individual-level correlation $\mathrm{cor}$. The leftmost column corresponds to no HPDH, and the middle row to no state-dependence. *OR* stands for the Odds-Ratio, that is $e^{\gamma}$. For the scenario with no HPDH or state-dependence (leftmost column, middle row): the relationship is flat, insensitive to the value of $\mathrm{cor}$ but only depends on the value of frailty ($\sigma_{\phi}$). The middle row corresponds to HPDH only: the effect of HPDH is to increase coefficient of variation of LRS, along with an increase in the value of $\mathrm{cor}$ or frailty ($\sigma_{\phi}$). When there is a trade-off between survival and breeding success ($cor<0$), the coefficient of variation is the smallest. However, when there is positive covariation between survival and breeding success ($cor>0$), it increased and the magnitude of the increased depends on frailty. The leftmost column corresponds to state-dependence only: state-dependence has little effect of the coefficient of variation of LRS. This insensitivity is expected as state-dependence with no heterogeneity corresponds to the case where individuals are identical with respect to breeding propensity.
