# Supplementary material for: Wolf in sheep's clothing: Model misspecification undermines tests of the neutral theory for life histories
Source: Ecol Evol. 2017 Apr 4;7(10):3348–61. doi: 10.1002/ece3.2874 (PMC5433986; doi:10.1002/ece3.2874)
Supplement: Supplementary file 3 [file ECE3-7-3348-s003.docx]

## Appendix S3: Graphical results for all simulation scenarios


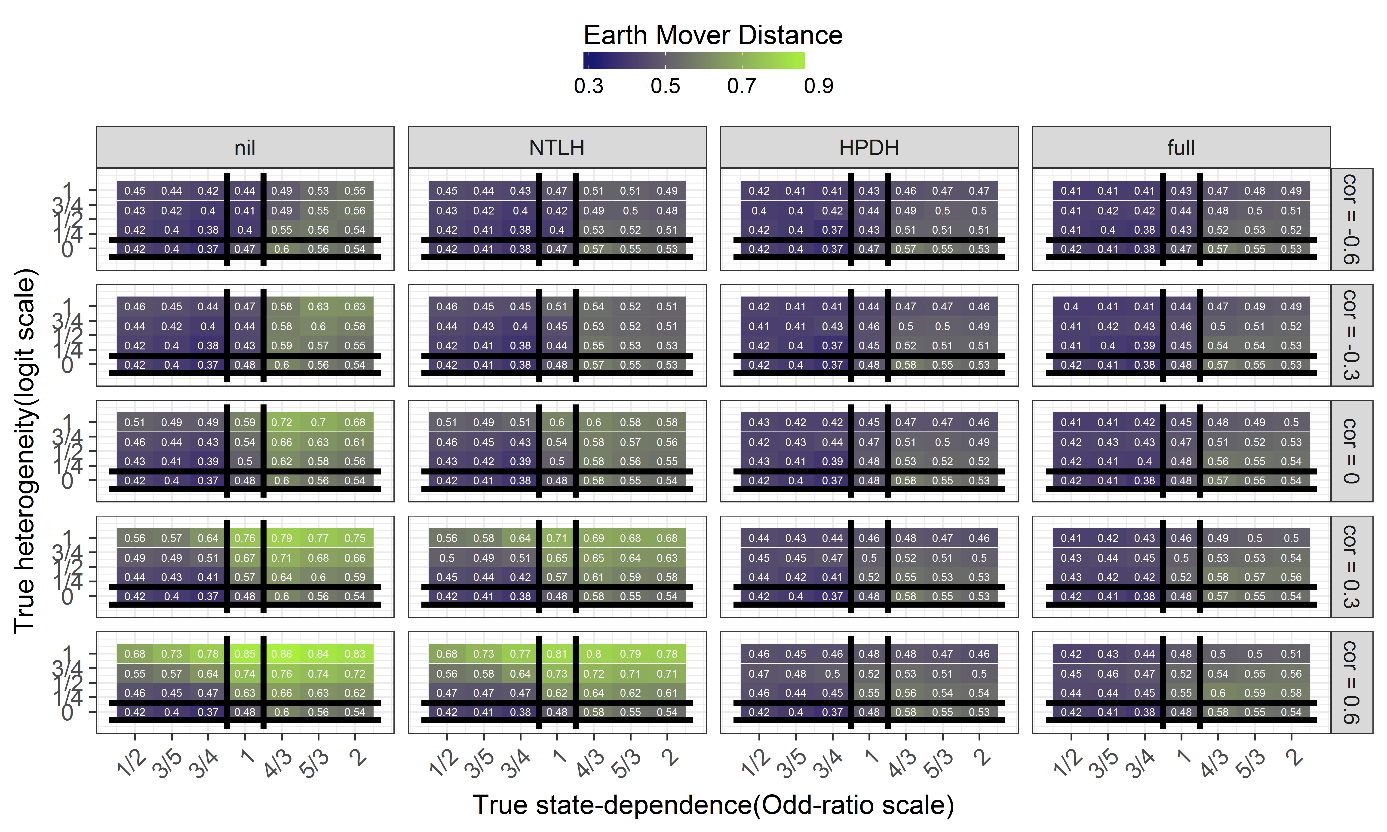


Figure S1: Tile-plots of the average estimated Earth Mover Distance (across 500 simulated datasets) between the observed and predicted distribution of LRS for each simulation scenario. True values of state-dependence ($e^{\gamma}$ on the Odds-Ratio scale) and HPDH ($\sigma_{\mathrm{repro}}$) are on the *x*− and *y*− axes respectively. Each row corresponds to a value of the individual-level correlation in HPDH used to simulate data. Each column corresponds to one of the four models used to analyze data. Vertical black lines bracket scenarios in which heterogeneity ($\mathcal{M}_{\mathrm{HPDH}}$) is the true data-generating mechanism. Horizontal black lines bracket scenarios in which state-dependence ($\mathcal{M}_{\mathrm{NTLH}}$) is the true data-generating mechanism. At the intersection, $\mathcal{M}_{\mathrm{nil}}$ is the true data-generating mechanism. Everywhere else, $\mathcal{M}_{\mathrm{full}}$ is the true data-generating mechanism. The true data-generating model should have the smallest Earth Mover distance. Actual values (rounded to the nearest integer) are displayed on each tile.


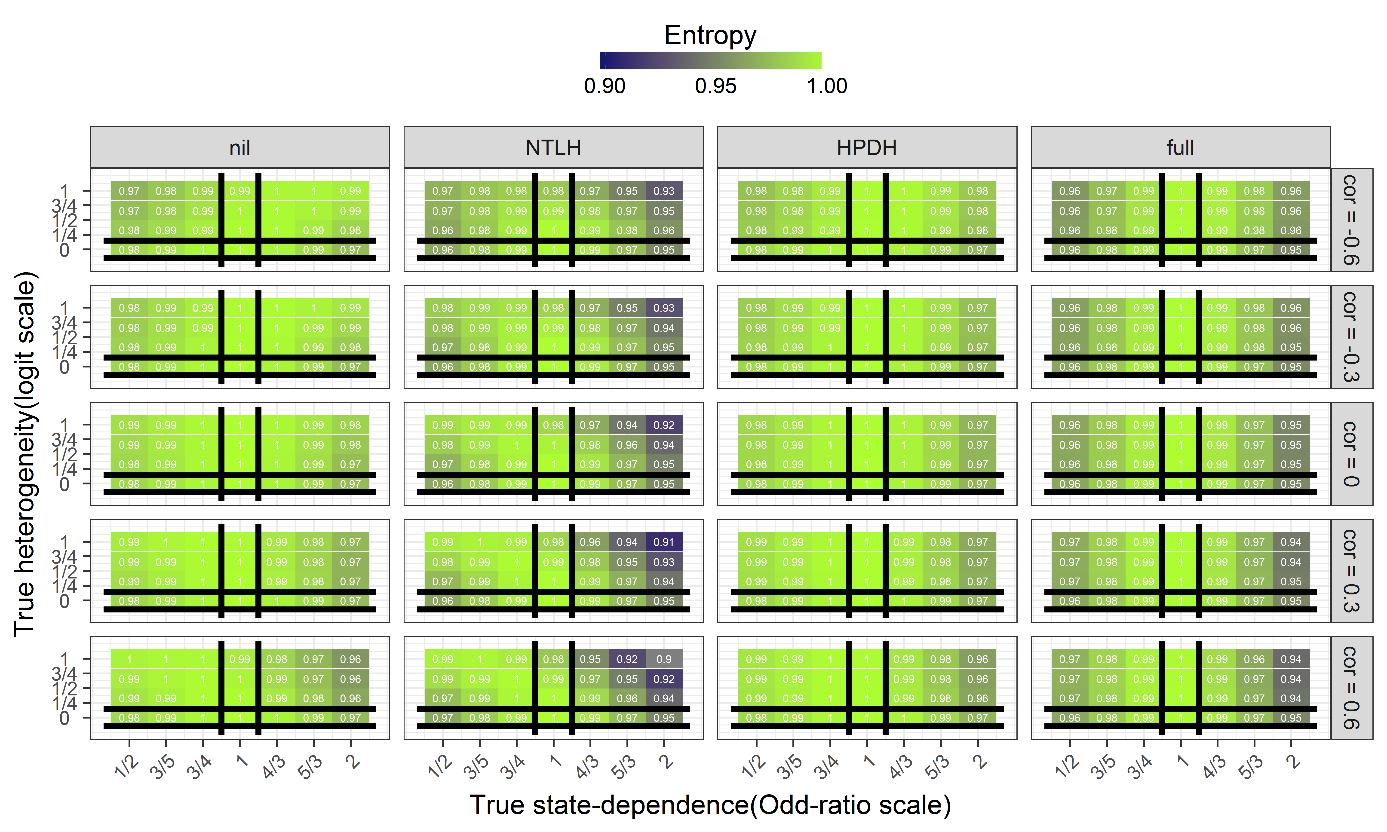


Figure S2: Tile-plots of the average estimated scaled entropy (across 500 simulated datasets) in each simulation scenario. True values of state-dependence ($e^{\gamma}$ on the Odds-Ratio scale) and HPDH ($\sigma_{\mathrm{repro}}$) are on the *x*− and *y*− axes respectively. Each row corresponds to a value of the individual-level correlation in HPDH used to simulate data. Each column corresponds to one of the four models used to analyze data. Vertical black lines bracket scenarios in which $\mathcal{M}_{\mathrm{HPDH}}$ is the true data-generating mechanism. Horizontal black lines bracket scenarios in which $\mathcal{M}_{\mathrm{NTLH}}$ is the true data-generating mechanism. At the intersection, $\mathcal{M}_{\mathrm{nil}}$ is the true data-generating mechanism. Everywhere else, $\mathcal{M}_{\mathrm{full}}$ is the true data-generating mechanism. Actual values (rounded to the nearest integer) are displayed on each tile.


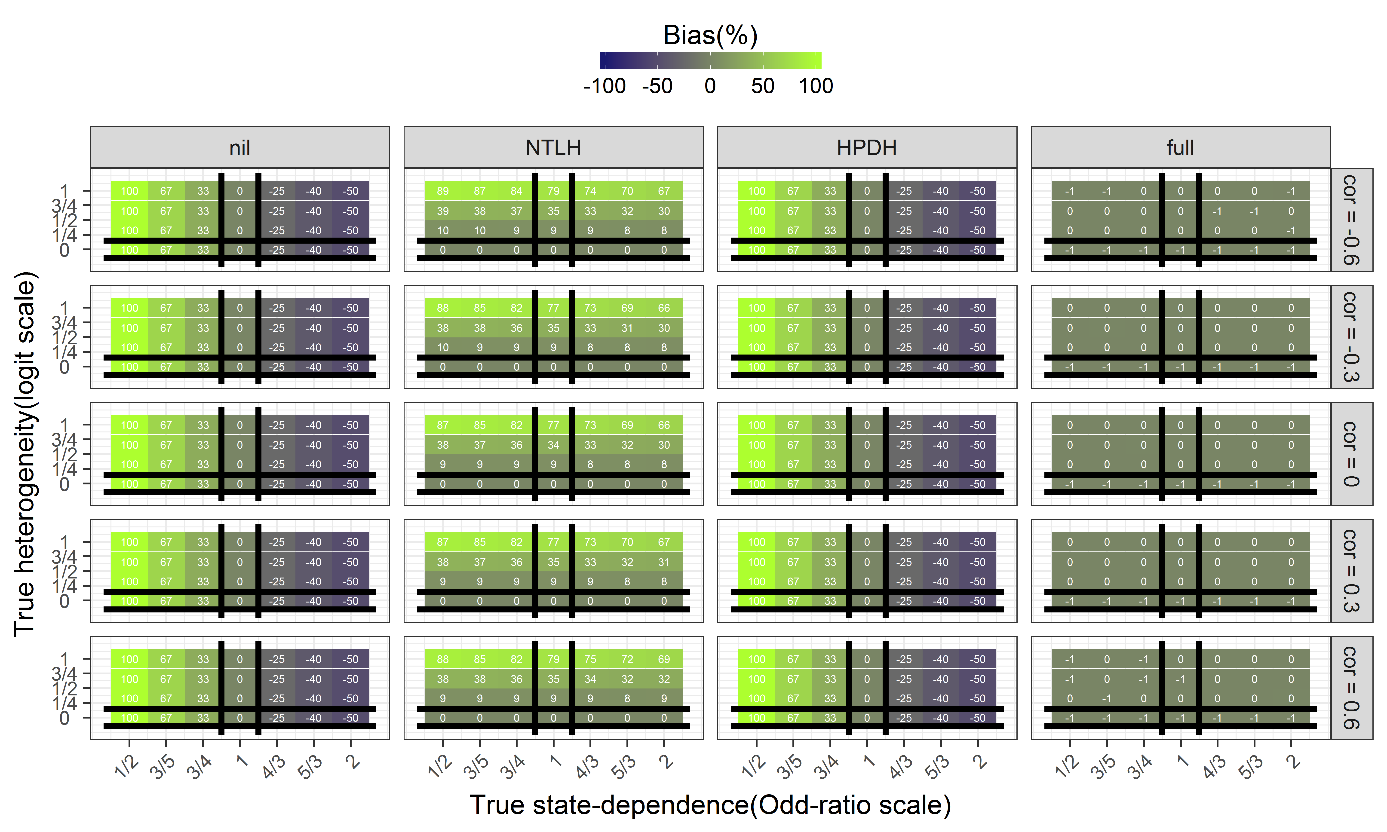


Figure S3: Tile-plots of the average bias in estimated state-dependence $\hat{\gamma}$ (across 500 simulated datasets) for each simulation scenario. True values of state-dependence ($e^{\gamma}$ on the Odds-Ratio scale) and HPDH ($\sigma_{\mathrm{repro}}$) are on the *x*− and *y*− axes respectively. Each row corresponds to a value of the individual-level correlation in HPDH used to simulate data. Each column corresponds to one of the four models used to analyze data. Vertical black lines bracket scenarios in which $\mathcal{M}_{\mathrm{HPDH}}$ is the true data-generating mechanism. Because $\mathcal{M}_{\mathrm{HPDH}}$ excludes $\gamma$, estimates ($\hat{\gamma}$) are by definition exactly 0. Horizontal black lines bracket scenarios in which $\mathcal{M}_{\mathrm{NTLH}}$ is the true data-generating mechanism. At the intersection, $\mathcal{M}_{\mathrm{nil}}$ is the true data-generating mechanism. Everywhere else, $\mathcal{M}_{\mathrm{full}}$ is the true data-generating mechanism. Estimates from the true data-generating model should have no bias on average. Actual bias values (rounded to the nearest integer) are displayed on each tile. Biases larger than 100% in magnitude were capped at 100%.


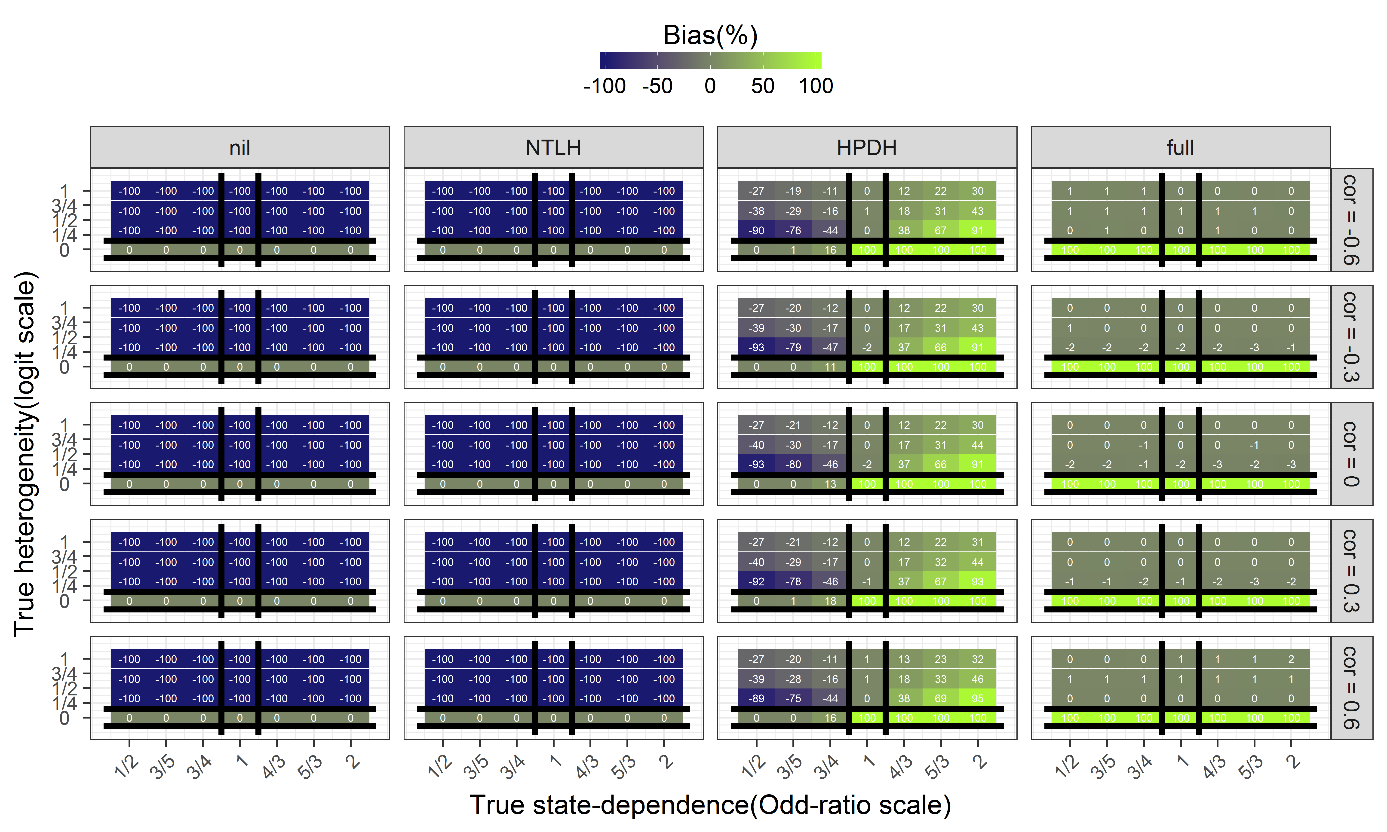


Figure S4: Tile-plots of the average bias in estimated HPDH $\hat{\sigma}_{\mathrm{repro}}$ (across 500 simulated datasets) for each simulation scenario. True values of state-dependence ($e^{\gamma}$ on the Odds-Ratio scale) and HPDH ($\sigma_{\mathrm{repro}}$) are on the *x*− and *y*− axes respectively. Each row corresponds to a value of the individual-level correlation in HPDH used to simulate data. Each column corresponds to one of the four models used to analyze data. Vertical black lines bracket scenarios in which $\mathcal{M}_{\mathrm{HPDH}}$ is the true data-generating mechanism. Horizontal black lines bracket scenarios in which $\mathcal{M}_{\mathrm{NTLH}}$ is the true data-generating mechanism. Because $\mathcal{M}_{\mathrm{NTLH}}$ excludes $\sigma_{\mathrm{repro}}$, estimates ($\hat{\sigma}_{\mathrm{repro}}$) are by definition exactly 0. At the intersection, $\mathcal{M}_{\mathrm{nil}}$ is the true data-generating mechanism. Everywhere else, $\mathcal{M}_{\mathrm{full}}$ is the true data-generating mechanism. Estimates from the true data-generating model should have no bias on average. Actual bias values (rounded to the nearest integer) are displayed on each tile. Biases larger than 100% in magnitude were capped at 100%.


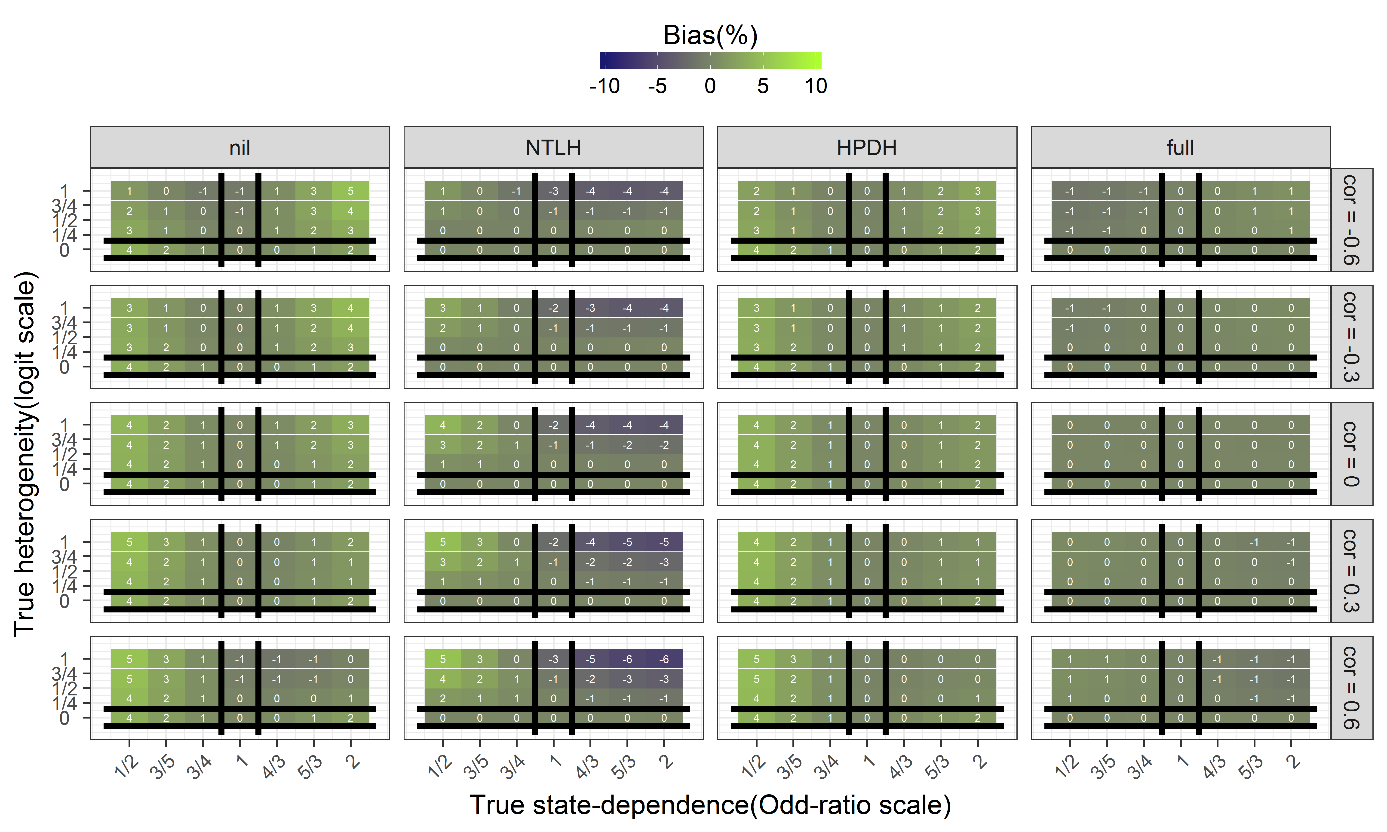


Figure S5: Tile-plots of the average bias in estimated within-individual variance ($\hat{\sigma}_{\mathrm{within}}^{2}$) for a 01 trajectory in breeding success of an average individual for each simulation scenario. True values of state-dependence ($e^{\gamma}$ on the Odds-Ratio scale) and HPDH ($\sigma_{\mathrm{repro}}$) are on the *x*− and *y*− axes respectively. Each row corresponds to a value of the individual-level correlation in HPDH used to simulate data. Each column corresponds to one of the four models used to analyze data. Vertical black lines bracket scenarios in which $\mathcal{M}_{\mathrm{HPDH}}$ is the true data-generating mechanism. Horizontal black lines bracket scenarios in which $\mathcal{M}_{\mathrm{NTLH}}$ is the true data-generating mechanism. At the intersection, $\mathcal{M}_{\mathrm{nil}}$ is the true data-generating mechanism. Everywhere else, $\mathcal{M}_{\mathrm{full}}$ is the true data-generating mechanism. Estimates from the true data-generating model should have no bias on average. Since variances are additive, any bias can blow up with the large sample considered in our simulations of a panel of 1*,*000 individuals. Actual bias values (rounded to the nearest integer) are displayed on each tile.


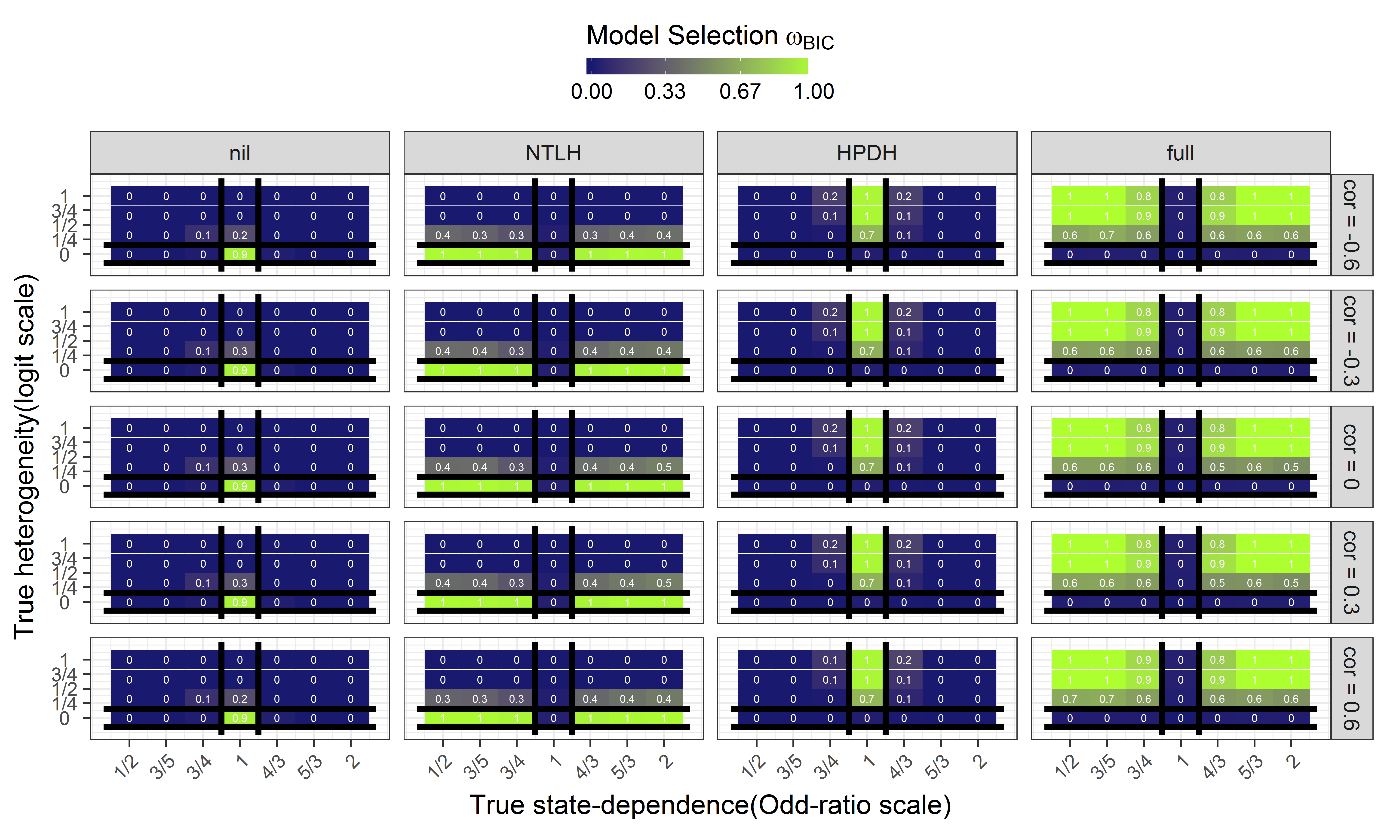


Figure S6: Tile-plots of the mean estimated $\hat{\omega}_{\mathrm{BIC}}$ (across 500 simulated datasets) for each model and each simulation scenario. True values of state-dependence ($e^{\gamma}$ on the Odds-Ratio scale) and HPDH ($\sigma_{\mathrm{repro}}$) are on the *x*− and *y*− axes respectively. Each row corresponds to a value of the individual-level correlation in HPDH used to simulate data. Each column corresponds to one of the four models used to analyze data. Vertical black lines bracket scenarios in which $\mathcal{M}_{\mathrm{HPDH}}$ is the true data-generating mechanism. Horizontal black lines bracket scenarios in which $\mathcal{M}_{\mathrm{NTLH}}$ is the true data-generating mechanism. At the intersection, $\mathcal{M}_{\mathrm{nil}}$ is the true data-generating mechanism. Everywhere else, $\mathcal{M}_{\mathrm{full}}$ is the true data-generating mechanism. The best predictive model, which would be selected for inference, has the largest weight. Actual values (rounded to one decimal) are displayed on each tile.


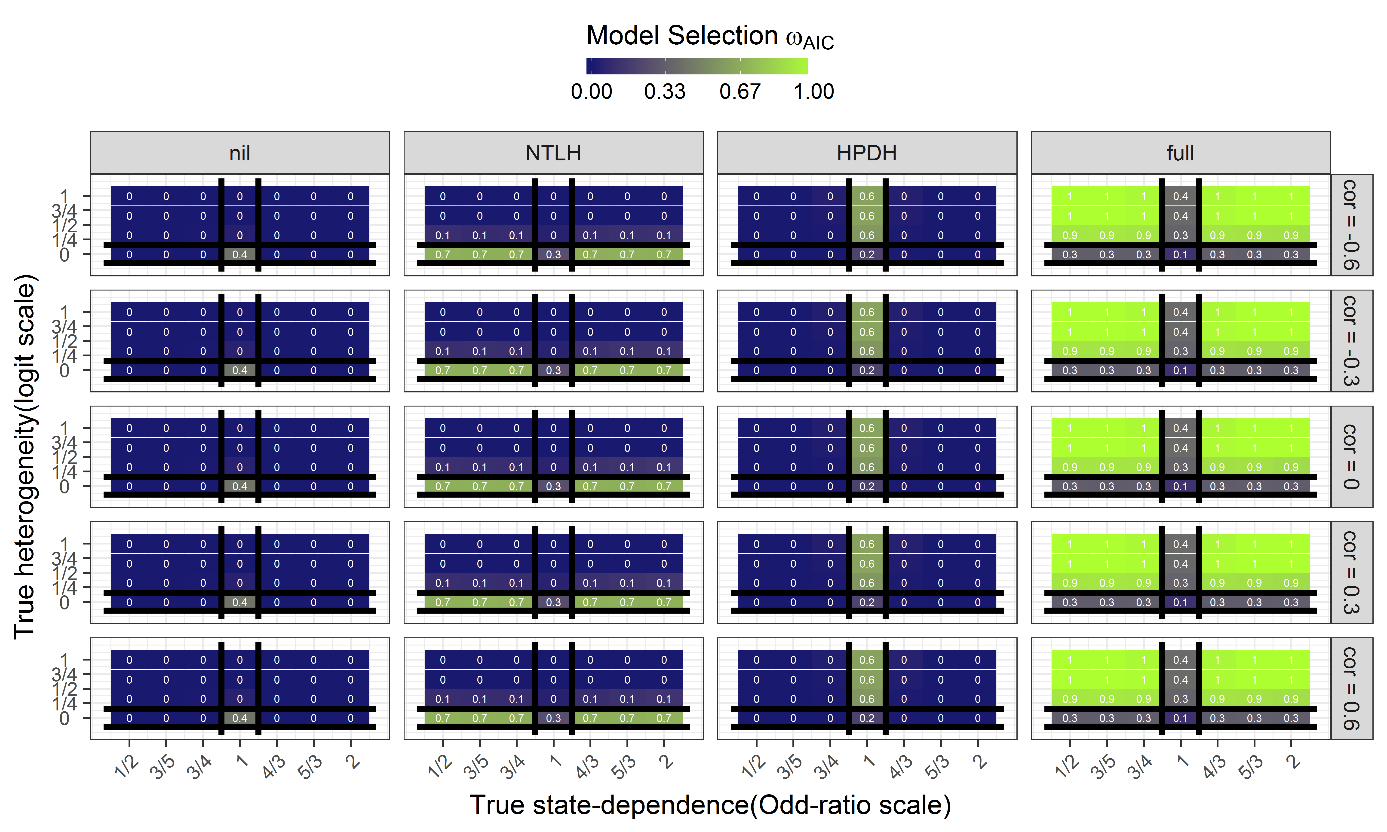


Figure S7: Tile-plots of the mean estimated $\hat{\omega}_{\mathrm{AIC}}$ (across 500 simulated datasets) for each model and each simulation scenario. True values of state-dependence ($e^{\gamma}$ on the Odds-Ratio scale) and HPDH ($\sigma_{\mathrm{repro}}$) are on the *x*− and *y*− axes respectively. Each row corresponds to a value of the individual-level correlation in HPDH used to simulate data. Each column corresponds to one of the four models used to analyze data. Vertical black lines bracket scenarios in which $\mathcal{M}_{\mathrm{HPDH}}$ is the true data-generating mechanism. Horizontal black lines bracket scenarios in which $\mathcal{M}_{\mathrm{NTLH}}$ is the true data-generating mechanism. At the intersection, $\mathcal{M}_{\mathrm{nil}}$ is the true data-generating mechanism. Everywhere else, $\mathcal{M}_{\mathrm{full}}$ is the true data-generating mechanism. The best predictive model, which would be selected for inference, has the largest weight. Actual values (rounded to one decimal) are displayed on each tile.


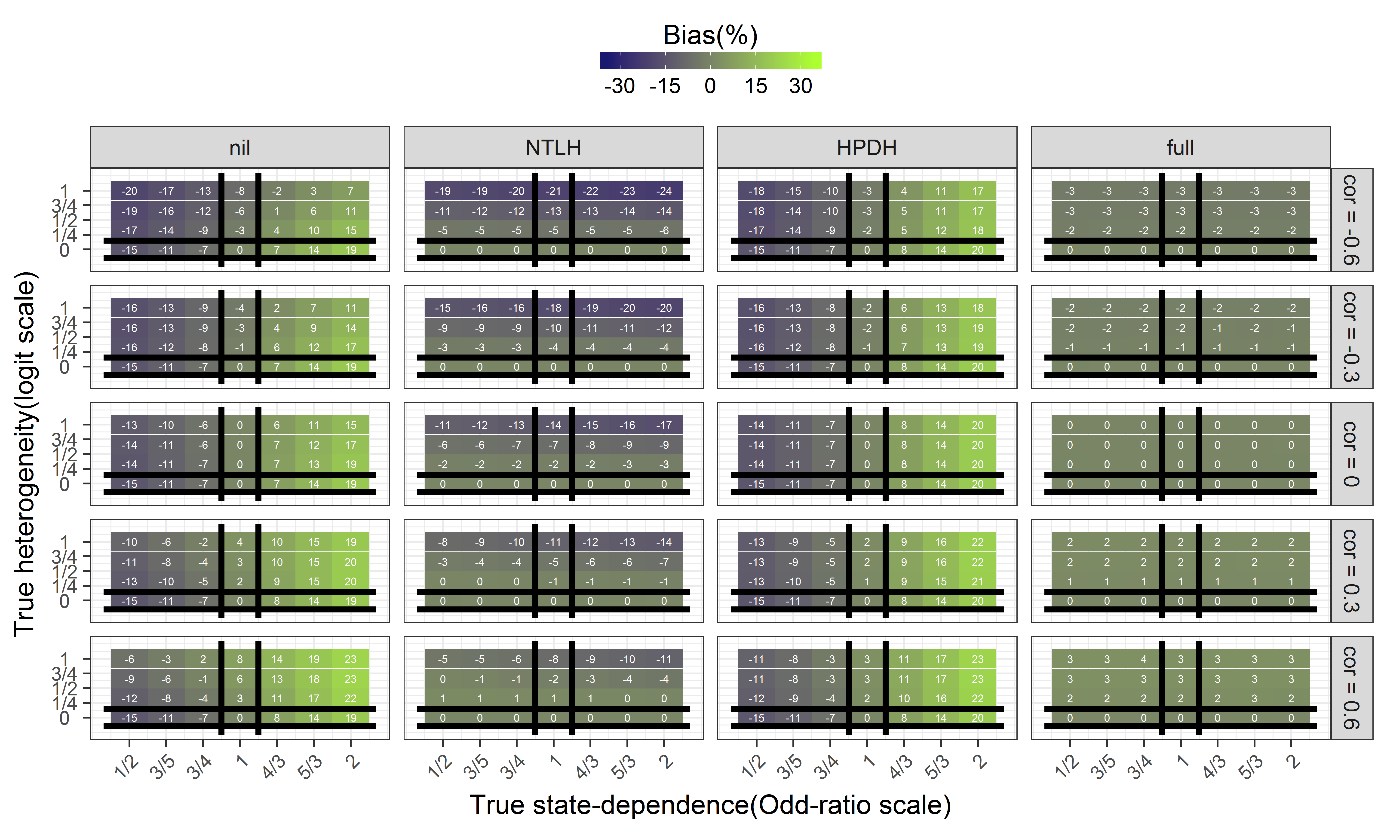


Figure S8: Tile-plots of the average bias in the estimated intercept $\hat{\mu}$ (across 500 simulated datasets) for each simulation scenario. True values of state-dependence ($e^{\gamma}$ on the Odds-Ratio scale) and HPDH ($\sigma_{\mathrm{repro}}$) are on the *x*− and *y*− axes respectively. Each row corresponds to a value of the individual-level correlation in HPDH used to simulate data. Each column corresponds to one of the four models used to analyze data. Vertical black lines bracket scenarios in which $\mathcal{M}_{\mathrm{HPDH}}$ is the true data-generating mechanism. Horizontal black lines bracket scenarios in which $\mathcal{M}_{\mathrm{NTLH}}$ is the true data-generating mechanism. At the intersection, $\mathcal{M}_{\mathrm{nil}}$ is the true data-generating mechanism. Everywhere else, $\mathcal{M}_{\mathrm{full}}$ is the true data-generating mechanism. Estimates from the true data-generating model should have no bias on average. Actual bias values are displayed on each tile.
