# Supplementary material for: Wolf in sheep's clothing: Model misspecification undermines tests of the neutral theory for life histories
Source: Ecol Evol. 2017 Apr 4;7(10):3348–61. doi: 10.1002/ece3.2874 (PMC5433986; doi:10.1002/ece3.2874)
Supplement: Supplementary file 4 [file ECE3-7-3348-s004.docx]

## Appendix S4: Model Selection - Some Methodological Considerations

Both $\mathcal{M}_{\mathrm{HPDH}}$ and $\mathcal{M}_{\mathrm{NTLH}}$ are simpler versions of $\mathcal{M}_{\mathrm{full}}$, but the direct comparison of $\mathcal{M}_{\mathrm{NTLH}}$, which is a generalized linear model, and of $\mathcal{M}_{\mathrm{HPDH}}$, which is a generalized linear mixed model, can be involved:

1. testing the nil hypothesis whether true state-dependence *γ* is zero is straightforward with a Likelihood Ratio Test of 1 degree of freedom;
2. testing for a nil variance involves a Likelihood Ratio Test with an equal mixture of 0 and 1 degree of freedom (Bolker et al., 2009; Gimenez and Choquet, 2010); but
3. simultaneous model selection of fixed and random effects is an open field of research in statistics (Delattre et al., 2014; Ibrahim et al., 2011).

Depending on sample size and the number of fixed effects, maximum likelihood estimates of variance parameters may be biased downward (Skrondal and Rabe-Hesketh, 2004, chapter 6, section 6). Downwardly biased estimates would result in too conservative statistical tests of $\sigma_{\mathrm{repro}}=0$ and an underestimation of the prevalence of HPDH. Restricted maximum likelihood estimates, the default option in lme4 (Bates et al. 2013), are preferred for estimating variance components in generalized linear mixed models (Bolker et al., 2009).

Model selection of the fixed effects structure, that is testing whether true state-dependence $\gamma$ is zero, is generally viewed as inappropriate with restricted maximum likelihood (Bolker et al. 2009; Russell, 2011, chapter 9). Yet Gurka (2006) documented with a Monte Carlo study that restricted maximum likelihood could be used to select the correct mean structure in linear mixed models. Our simulation results showed that standard information theoretic tools such as $\hat{\omega}_{\mathrm{AIC}}$ (Burnham and Anderson, 2002; Burnham and White, 2002) or $\hat{\omega}_{\mathrm{BIC}}$ (Link and Barker, 2009), could be used with restricted maximum likelihood to infer the correct data-generating mechanism: they gave in fact the same results as Likelihood Ratio Tests (not shown). However, we should not lose sight that we placed ourselves in a favorable situation with large sample size and long trajectories, and made the crude approximation that there was only one additional (variance) parameter to estimate instead of estimating the effective number of parameters in $\mathcal{M}_{\mathrm{HPDH}}$ or $\mathcal{M}_{\mathrm{full}}$ (see also Delattre et al., 2014).

The difficult question of model selection can have profound consequences on inference. There are alternative to information criteria when selecting a model. The idea to select a model based on its ability to predict a salient summary statistic of the data (for example LRS) is behind the idea of posterior predictive checks in Bayesian model selection (Chambert et al., 2014; Gelman et al., 1996). These checks generalize classical hypothesis testing by averaging over the posterior distribution of unknown parameters rather than using point estimates, but require careful consideration of the test statistic with respect to the inferential goal (Gelman, 2013; Gelman et al., 1996). The idea behind comparing predicted and observed LRS distribution is similar: the goal is to infer the data-generating mechanism of individual life histories, not predicting accurately population-level LRS; although the latter is assumed necessary for the former. We showed that this assumption is unwarranted: using LRS to reveal systematic differences between models implying different demographic processes is inappropriate. This stems from a disjunction between pattern and process, which is a recurring theme within ecology (Chave et al., 2002; Leigh, 2007; Warren II et al., 2011).

References

Bates, D., M. Maechler, B. Bolker, and S. Walker, 2013. lme4: Linear mixed-effects models using Eigen and S4. <http://CRAN.R-project.org/package=lme4>.

Bolker, B., M. Brooks, C. Clark, S. Geange, J. Poulsen, M. Stevens, and J.-S. White. 2009. Generalized Linear Mixed Models: a Practical Guide for Ecology and Evolution. Trends in Ecology and Evolution 24:127–135.

Burnham, K., and D. Anderson. 2002. Model Selection and Multimodel Inference. A Practical Information-Theoretic Approach. 2^nd^ edition. Springer. 488 pages. New York, USA.

Burnham, K. P., and G. C. White. 2002. Evaluation of some random effects methodology applicable to bird ringing data. Journal of Applied Statistics 29:245–264.

Chambert, T., J. Rotella, and J. Higgs. 2014. Use of Posterior Predictive Checks as an Inferential Tool for Investigating Individual Heterogeneity in Animal Population Vital Rates. Ecology and Evolution 4:1389–1397.

Chave, J., H. Muller-Landau, and S. Levin. 2002. Comparing Classical Community Models: Theoretical Consequences for Patterns of Diversity. The American Naturalist 159:1–23.

Delattre, M., M. Lavielle, and M. Poursat. 2014. A Note on BIC in Mixed-Effects Models. Electronic Journal of Statistics 8:456–475.

Gelman, A. 2013. Two Simple Examples for Understanding Posterior p-values whose Distributions Are Far from Uniform. Electronic Journal of Statistics 7:2595–2602.

Gelman, A., X.-L. Meng, and H. Stern. 1996. Posterior Predictive Assessment of Model Fitness via Realized Discrepancies. Statistica Sinica 6:733–807.

Gimenez, O., and R. Choquet. 2010. Individual Heterogeneity in Studies on Marked Animals using Numerical Integration: Capture–Recapture Mixed Models. Ecology 91:951–957.

Gurka, M. 2006. Selecting the Best Linear Mixed Model Under REML. The American Statistician 60:19–26.

Ibrahim, J., H. Zhu, R. Garcia, and R. Guo. 2011. Fixed and Random Effects Selection in Mixed Effects Models. Biometrics 67:495–503.

Leigh, E. G. 2007. Neutral Theory: a Historical Perspective. Journal of Evolutionary Biology 20:2075–2091.

Link, W., and R. Barker, 2009. Bayesian Inference with Ecological Applications, Chapter 12 - Individual Fitness, pages 271–286. 1^st^ edition. 354 pages. Associated Press. London, UK.

Russell, B. 2011. Maximum Likelihood Estimation and Inference - With Examples in R, SAS and ADMB. 1^st^ edition. Wiley-Blackwell. 376 pages. Chichester, UK.

Skrondal, A., and S. Rabe-Hesketh. 2004. Generalized Latent Variable Modelling - Multilevel, Longitudinal and Structural Equation Models. 1^st^ edition. Chapman & Hall/ CRC. 528 pages. Boca Raton, Florida, USA.

Warren II, R., D. Skelly, O. Schmitz, and M. Bradford. 2011. Universal Ecological Patterns in College Basketball Communities. PLoS ONE 6:e17342.
